# Decoding lysophosphatidic acid signaling in physiology and disease: mapping the multimodal and multinodal signaling networks

**DOI:** 10.1038/s41392-025-02423-4

**Published:** 2025-10-10

**Authors:** Revathy Nadhan, Karthik Nath, Sneha Basu, Ciro Isidoro, Yong Sang Song, Danny N. Dhanasekaran

**Affiliations:** 1 Stephenson Cancer Center, The University of Oklahoma Health Sciences Center, Oklahoma City, OK USA; 2 Nvidia Corporation, 2788 San Tomas Expressway, Santa Clara, CA USA; 3 Department of Pathology, The University of Oklahoma Health Sciences Center, Oklahoma City, OK USA; 4 Laboratory of Molecular Pathology and NanoBioImaging, Department of Health Sciences, Università del Piemonte Orientale, Novara, Italy; 5 Department of Obstetrics and Gynecology, Cancer Research Institute, College of Medicine, Seoul National University, Seoul, Korea; 6 Department of Cell Biology, The University of Oklahoma Health Sciences Center, Oklahoma City, OK USA

**Keywords:** Cancer models, Translational research

## Abstract

Lysophosphatidic acid (LPA) signaling has emerged as a central regulatory axis in both normal physiology and disease, orchestrating diverse cellular processes such as proliferation, survival, migration, immune modulation, and tissue remodeling. Originally identified as a bioactive lipid that regulates smooth muscle contraction and vascular tone, LPA has since emerged as a pleiotropic signaling molecule implicated in multiple physiological systems and a wide spectrum of pathological states. These include cancer, neurodegenerative disorders, cardiovascular and metabolic syndromes, inflammatory conditions, and fibrotic diseases. Elevated LPA levels, overexpression of autotaxin (ATX), and aberrant activation of LPA receptors (LPARs) contribute to disease initiation and progression, positioning the LPA axis as both a diagnostic biomarker and a promising therapeutic target. This review describes the multimodal and multinodal organization of the LPA signaling network, detailing upstream biosynthesis, receptor diversity, and downstream effectors across diverse organ systems. Therapeutic strategies targeting ATX, LPARs, and intracellular mediators are critically assessed, along with a review of ongoing and emerging clinical trials. Furthermore, we introduce a context-aware AI-based neural network model to simulate LPA signaling dynamics, providing a framework for predictive modeling and personalized therapeutic interventions. By integrating mechanistic insights with adaptive computational frameworks, this review positions the LPA axis as a powerful and versatile target for systems biology-guided precision medicine strategies in both health and disease.

## Introduction

Lysophosphatidic acid (LPA) has transitioned from a relatively obscure phospholipid intermediate to a central regulator of diverse physiological and pathological processes.^1–4^ As a bioactive lipid mediator, LPA signals primarily through six G protein-coupled receptors (GPCRs) termed as LPA receptors, LPAR1–6, which modulate key cellular functions such as proliferation, survival, motility, cytoskeletal remodeling, and immune regulation.^1,3,5^ Upon binding to its receptors on the cell membrane, LPA activates Gα proteins, initiating downstream pathways such as mitogen-activated protein kinase kinase (MEK)/extracellular signal-regulated kinase (ERK), phosphoinositide 3-kinase (PI3K)/Akt, and Rho/Rho kinase (ROCK).^1–4^

Over the past five decades, landmark discoveries in LPA biology have not only deepened our understanding of lipid signaling but also placed it at the crossroads of developmental biology, systems physiology, and disease pathogenesis.^1–4^ While initially studied in the context of platelet physiology and wound healing, LPA signaling is now recognized as a contributor to fibrosis, neurodegenerative and psychiatric disorders, cardiovascular diseases, metabolic dysfunctions, and autoimmune inflammation.^2,5–7^ These pleiotropic effects arise from a complex multimodal and multinodal network involving pathways such as the PI3K/Akt, MAPK/ERK, Rho/ROCK, EGFR, Wnt/β-catenin, and Hippo-YAP pathways.^8–14^

This review presents a comprehensive analysis of LPA signaling, tracing its historical trajectory and decoding its molecular functions across physiological and disease contexts. We examine the core and auxiliary signaling pathways affected by LPA, highlight its regulatory mechanisms, and explore its functional roles from development and homeostasis to complex pathologies, including cancer and cardiovascular, neurodegenerative, metabolic, autoimmune, and inflammatory diseases. In addition, we assess the therapeutic landscape by examining pharmacological inhibitors, current clinical trials, and emerging drug targets. We also highlight the growing integration of artificial intelligence (AI)-based systems biology tools for modeling LPA circuits and designing precision interventions. Together, these findings position LPA signaling as a versatile and targetable axis in translational medicine.

## Milestones in LPA discovery and research

**Fig. 1:**
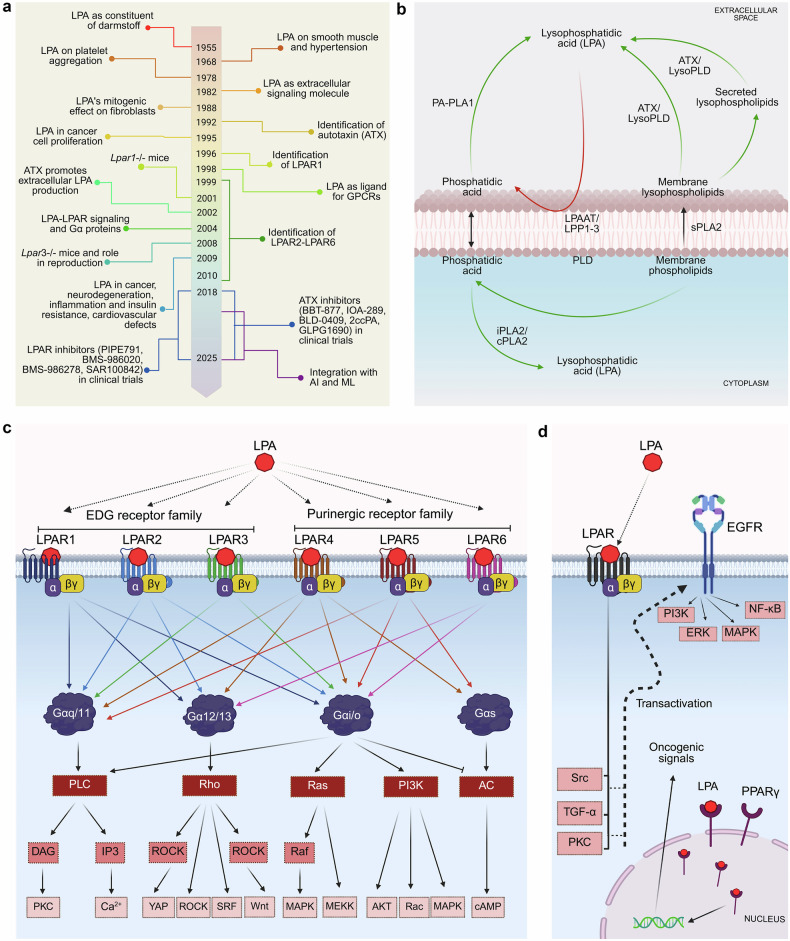
LPA discovery, signaling and homeostasis. The figure depicts **a** the chronological milestones in LPA research; **b** the enzymatic pathways involved in LPA synthesis (green arrow lines) and degradation (red arrow lines) that maintain its homeostasis; **c** the classical LPA signaling pathways downstream of six LPA receptors, categorized into the EDG and purinergic receptor families, associated G proteins and downstream effectors and second messenger molecules that promote diverse pathophysiological responses; and **d** the noncanonical LPA signaling mechanisms that include the PPARγ-mediated pathway and transactivation of RTKs [AC adenylyl cyclase, AKT serine threonine kinase, AI artificial intelligence, ATX autotaxin, Ca2 + - calcium, cAMP cyclic AMP, cPLA2 Ca2 + -dependent cytosolic PLA2, DAG diacylglycerol, EDG endothelial differentiation gene, EGFR epidermal growth factor receptor, ERK extracellular signal-regulated kinase, IP3 inositol 1,4,5-trisphosphate, iPLA2 inducible PLA2, LPA lysophosphatidic acid, LPAAT LPA Acyltransferases, LPAR LPA receptor, LPP—The figure was created via BioRender.com under an academic license

LPA was initially regarded as a minor phospholipid intermediate, but over the past five decades, it has emerged as a central signaling molecule with broad physiological and pathological relevance. This evolution reflects a series of pivotal discoveries, from its early identification as a mitogenic lipid to recent advances in receptor biology, enzymatic biosynthesis, and computational modeling (Fig. 1a).

### Early biochemical insights (1960s–1980s)

LPA was first identified in the 1960s as a serum lysophospholipid that induces smooth muscle contraction and promotes platelet aggregation,^15–17^ establishing the concept that lipids can serve as extracellular signaling molecules.^18^ LPA is present in serum, and its production during blood clotting points to its broader physiological roles in tissue repair and inflammatory responses.^16,19,20^ Furthermore, its mitogenic effects on fibroblasts and other cell types suggest its potential role in cellular proliferation and cancer biology.^21–23^

### Receptor discovery and signal transduction (1990s)

A turning point came in 1996 with the cloning of the first LPA receptor, LPAR1 (EDG2),^24^ identifying LPA as a GPCR ligand. Subsequent identification of LPAR2 through LPAR6 expanded its signaling repertoire.^25,26^ These receptors couple with Gαi, Gαq, Gα12/13, and Gαs, activating diverse pathways, including the PI3K/Akt, MEK/ERK, and Rho/ROCK pathways.

### Autotaxin and LPA biosynthesis (Late 1990s–2000s)

Another key discovery was the identification of autotaxin (ATX)/ENPP2 as the enzyme responsible for the generation of extracellular LPA from lysophosphatidylcholine (LPC).^27^ Initially, isolated as a tumor cell motility factor,^28^ ATX was later shown to possess lysophospholipase D activity, thereby directly linking it to LPA biosynthesis.^29^ This discovery clarified the enzymatic source of bioactive LPA in the extracellular milieu and introduced ATX as a promising therapeutic target in both cancer and inflammatory diseases.

### Receptor knockout and in vivo function (2000s–2010s)

During the 2000s and 2010s, functional insights into LPA signaling significantly advanced through the development of LPAR knockout mice. These genetically modified models revealed receptor-specific roles in development and physiology. For example, Lpar1⁻/⁻ mice presented defects in neurodevelopment, vascular integrity, and auditory processing,^30^ whereas Lpar3⁻/⁻ females presented impaired embryo implantation, highlighting the tissue-specific roles of LPARs in vivo.^31,32^

### Pathophysiological roles and disease associations (2010s)

By the 2010s, the relevance of LPA signaling to human disease was increasingly recognized. Elevated LPA levels and dysregulated receptor expression are linked to cancer, fibrosis, neurodegeneration, cardiovascular, and metabolic disorders.^1–4^ LPA has been shown to influence immune cell trafficking, angiogenesis, nociception, and tissue fibrosis, reinforcing its role in chronic inflammation and therapeutic resistance.^33–35^

### Therapeutic targeting and clinical translation (2010s–present)

Translational interest in the LPA axis grew during this decade, culminating in the development of pharmacologic inhibitors targeting both ATX (e.g., GLPG1690) and LPARs (e.g., BMS-986020).^36–40^ Several candidates have entered clinical trials for the treatment of fibrotic diseases and cancer, confirming the druggable potential of the ATX–LPA axis.^36,38,40–43^

### Integration with AI and precision medicine (2020 onward)

LPA research has entered a computational era, with AI and machine learning applied to map disease-specific LPA networks, simulate therapeutic responses, and develop precision interventions. These tools enable dynamic, predictive models of LPA signaling for translational applications.

This progression, from early biochemical findings to AI-driven modeling, illustrates how LPA biology has matured into a translationally relevant field. The following sections dissect the components and mechanisms that define this multimodal and multinodal signaling network.

## Components of LPA signaling

LPA acts through a complex signaling network anchored by its six receptors (LPAR1–6), each linked to distinct G protein subtypes that mediate downstream effects on proliferation, motility, survival, and differentiation.

### LPA biosynthesis, degradation, and signaling

LPA is a structurally simple phospholipid with a single fatty acid chain. It has a glycerol backbone with a sn-3 phosphate group, a hydroxyl group, and a sn-1 or sn-2 fatty acid chain. It is synthesized both intracellularly and extracellularly by diverse enzymes (Fig. 1b). Extracellularly, ATX (encoded by the *ENPP2* gene) is the primary enzyme that produces LPA from lysophospholipids, which are often anchored near LPARs via integrins.^44^ ATX, a lysophospholipase, removes the phosphate group from membrane-bound lysophospholipids to produce LPA.^45^ Soluble PLA2 (sPLA2) contributes to LPA synthesis by generating lysophospholipids from phosphatidylcholine, which ATX subsequently hydrolyzes.^46^ LPA is also synthesized from membrane-bound phosphatidic acid by phospholipase A1 (PLA1).^47^ Intracellularly, inducible PLA2 (iPLA2) and calcium-dependent cytosolic PLA2 (cPLA2) contribute to LPA synthesis from phosphatidic acid.^48^ LPA degradation is primarily mediated by lipid phosphate phosphatases 1-3 (LPPs1-3), which convert LPA to monoacylglycerol.^49^ LPA is also catabolized by LPA phosphatase type 6 (acid phosphatase 6 or ACP6) and lysophosphatidic acid phosphatase like protein 6 (PACPL1).^50^ In addition to these phosphatases, LPA acyltransferases (LPAATs) reesterify LPA to form phosphatidic acid.^51^

The balance of LPA synthesis and degradation regulated by the ATX‒LPA‒LPAR‒LPP nexus is critical for normal physiological processes.^52^ Dysregulation of LPA homeostasis through altered expression or activity of LPA, ATX, LPA, LPAR, or LPPs contributes to many diseases, including cancer, cardiovascular disorders, autoimmune disorders, neurological conditions and other inflammatory diseases.^53–56^

A distinctive hallmark of LPA signaling is its multimodal and multinodal architecture. LPA initiates diverse signaling modes, including the activation of GPCRs, transactivation of receptor tyrosine kinases, ion channel modulation, and nuclear receptor interactions, thereby orchestrating a wide spectrum of cellular functions. Downstream of its receptors (LPAR1–6), LPA engages multiple signaling nodes, such as PI3K, ERK, RhoA, and YAP, which serve as hubs that integrate and redistribute signals across interconnected pathways. This multimodal framework enables context-dependent signaling plasticity, equipping the LPA network to regulate complex phenotypes across both physiological and pathological conditions.

### LPARs

LPA signals through the activation of six LPARs^57,58^ that are coupled to one or more of the G proteins, namely, Gs, Gi, Go, Gq, G11, G12 and G13^57^ (Fig. 1c). LPAR1, LPAR2, and LPAR3 belong to the endothelial differentiation gene (EDG) family (known as EDG2, EDG4, and EDG7), whereas LPAR4, LPAR5, and LPAR6 belong to the purinergic GPCR family (known as P2Y9/GPR23, GPR92, and P2Y5).^59^ Each LPAR subtype exhibits a unique expression pattern and has the potential to couple with different types of G proteins, thereby activating distinct downstream signaling pathways. For example, LPAR1 can couple with Gαi, Gαq, or Gα12/13 in a tissue- and context-specific manner, highlighting its versatility in signaling.^57^ In addition to LPARs, peroxisome proliferator-activated receptor gamma (PPARγ), which is located in the nucleus, has also been reported to serve as an intracellular receptor for LPA.^60^

### Downstream effectors

LPARs couple with various heterotrimeric G proteins, including Gαs, Gαi, Gαq, Gα12, and Gα13 (Fig. 1c). Specific interactions with the Gα- and Gβγ-subunits primarily affect downstream signaling pathways and cellular responses. Thus, LPA-LPAR signaling pathways are primarily determined by the specific G protein subtypes engaged by the respective LPARs, dictating downstream signaling cascades and ultimately cellular responses, ranging from proliferation and survival to inflammation, immune responses, migration and differentiation.

LPAR activation of Gαs stimulates adenylate cyclase, increasing cyclic AMP (cAMP) levels, whereas activation of Gαi inhibits adenylate cyclase.^61^ Gαq activation leads to the stimulation of phospholipase C (PLC), resulting in the production of inositol trisphosphate (IP3) and diacylglycerol (DAG).^62^ Gα12 and Gα13 regulate the Rho family of GTPases, affecting the actin cytoskeleton and cell motility.^63^

LPAR1 couples with Gα12/13, Gαq/11 or Gαi/o to elicit downstream responses, mainly through the Akt, MAPK, PLC, or Rho pathways.^57^ LPAR2 also couples with Gα12/13, Gαq/11 or Gαi/o to elicit signaling responses mediated through the PI3K, PLC, MAPK, Rac, Rho, and Ras pathways.^1–4^ LPAR3 binds to Gαq/11 or Gαi/o, initiating MAPK and PLC signaling cascades.^1–4^ LPAR4 couples with Gα12/13, Gαi/o, Gαq/11 or Gαs, whereas LPAR5 primarily interacts with Gα12/13, Gαi/o, or Gαq/11.^1–4^ Although the specific role of Gβγ subunits following LPAR activation has not been thoroughly investigated, dissociated Gβγ subunits can potentiate signaling by GPCRs, playing a synergistic role in the diverse pathways activated by LPARs.

The cAMP and protein kinase A (PKA) pathway, which is traditionally associated with metabolic regulation, also plays a nuanced and context-dependent role in LPA signaling. LPA signaling regulates cAMP levels via two G proteins: Gαs, which stimulates adenylate cyclase to increase cAMP levels, and Gαi, which inhibits adenylate cyclase, reducing cAMP levels.^61^ Additionally, the LPAR5 receptor (also known as GPR92) has been shown to increase cAMP levels through the Gα12/13 and Gαq proteins.^64^

### Noncanonical signaling by LPA

LPA signaling extends beyond canonical G protein-coupled mechanisms, engaging alternative pathways that contribute to its complexity in signaling (Fig. 1d). One key mechanism is the transactivation of receptor tyrosine kinases (RTKs), such as epidermal growth factor receptor (EGFR), which activates the downstream Ras/ERK and PI3K/Akt pathways.^65–68^ These transactivation events amplify downstream signals and integrate LPA signaling with broader cellular networks driven by EGFR.

In addition to RTK transactivation, LPA can activate the nuclear receptor PPARγ, influencing the expression of genes related to lipid metabolism, inflammation, and cell proliferation.^1,5^ LPARs can also mediate atypical or G protein-independent signaling pathways. β-Arrestin proteins, which are activated by LPARs, act as scaffolding proteins that mediate receptor desensitization and alternative signaling, influencing cell migration and cytoskeletal reorganization.^50,69^

### Crosstalk with other pathways

LPA signaling involves extensive crosstalk with key pathways, such as the Wnt/β-catenin pathway, influencing EMT, proliferation, and migration.^12^ It also modulates the Hippo–YAP pathway via LPAR3-Gα13-Rho-ROCK signaling, which activates the PP1-mediated dephosphorylation of YAP. Activated YAP enhances the expression of amphiregulin (AREG), a ligand for EGFR, thereby initiating EGFR-driven oncogenic signaling.^13^

Receptor-level compensation adds another layer of regulation. Loss or inhibition of one LPAR (e.g., LPAR1) can lead to upregulation or enhanced activity of others (e.g., LPAR2 or LPAR3), maintaining downstream effects such as proliferation, migration, immune activation, and fibrogenesis.^70^ This functional interplay among LPARs underscores the complexity of LPA signaling targeting and highlights the importance of accounting for compensatory mechanisms when therapeutic strategies are designed.

### Spatiotemporal dynamics of LPA signaling

LPA signaling involves tightly regulated spatiotemporal dynamics that critically shape its roles in cancer, fibrosis, neurodegeneration, and immune disorders. These dynamics encompass time-sensitive receptor activation, context-dependent signaling, and feedback regulation, each of which contributes to disease-specific outcomes.

Temporally, LPA induces rapid activation of the MAPK, PI3K, and RhoA pathways, promoting cell migration and survival.^1,3,5,71^ However, prolonged stimulation triggers receptor desensitization, biased pathway activation, and adaptive feedback, altering signaling outputs. These time-dependent shifts modulate key pathological processes, including metastasis, fibrogenesis, and immune evasion.

Spatially, LPAR expression and function vary by tissue and disease context. LPAR1, for example, may increase proliferation in tumors but exert protective effects in fibrotic or neural tissues.^1,3,5,71^ Factors such as receptor localization, endocytosis, posttranslational modifications, and G-protein coupling influence signaling diversity and cellular responses.

LPA signaling is further shaped by feedback loops: ATX upregulation reinforces LPA production and pathological signaling, whereas lipid phosphate phosphatases (LPPs) degrade extracellular LPA to dampen signaling.^49,72^ Dysregulation of this feedback contributes to chronic inflammation, fibrosis, and tumorigenesis, and restoring this balance represents a promising therapeutic strategy.

### Multilevel regulation of LPA signaling

LPA signaling is dynamically regulated through transcriptional, epigenetic, and metabolic mechanisms, enabling adaptation to tissue-specific and pathological contexts.

#### Transcriptional regulation

The expression of core components, ATX/ENPP2, LPARs, and lipid-modifying enzymes is influenced by transcription factors activated by growth factors, cytokines, and stress. Hypoxia-inducible factors (HIFs) upregulate ENPP2 in the tumor microenvironment (TME), linking hypoxia to elevated LPA levels.^73^ NF-κB and STAT3 also promote LPAR expression in cancer and inflammation, creating a feedforward loop that enhances survival and immune evasion.^74^

#### Epigenetic regulation

DNA methylation and histone modifications modulate the chromatin accessibility of genes involved in LPA signaling. Promoter methylation of lipid phosphate phosphatases (LPPs) reduces LPA degradation in certain cancers.^37^ MicroRNAs such as miR-489 target ENPP2 and LPAR mRNAs, modulating LPA output.^75,76^

#### Metabolic regulation

LPA biosynthesis is closely tied to lipid metabolism. Substrate availability (e.g., LPC), altered PLA2 activity, and lipid droplet dynamics affect LPA production.^77^ Insulin and glucose signaling intersect with ATX expression and the phospholipid supply, suggesting that metabolic states such as obesity or insulin resistance directly modulate the LPA axis.^78–80^

Collectively, these regulatory layers underscore LPA signaling as a responsive, multitiered network, providing actionable targets in diseases driven by its dysregulation.

## LPA-LPAR signaling in normal physiology

**Fig. 2:**
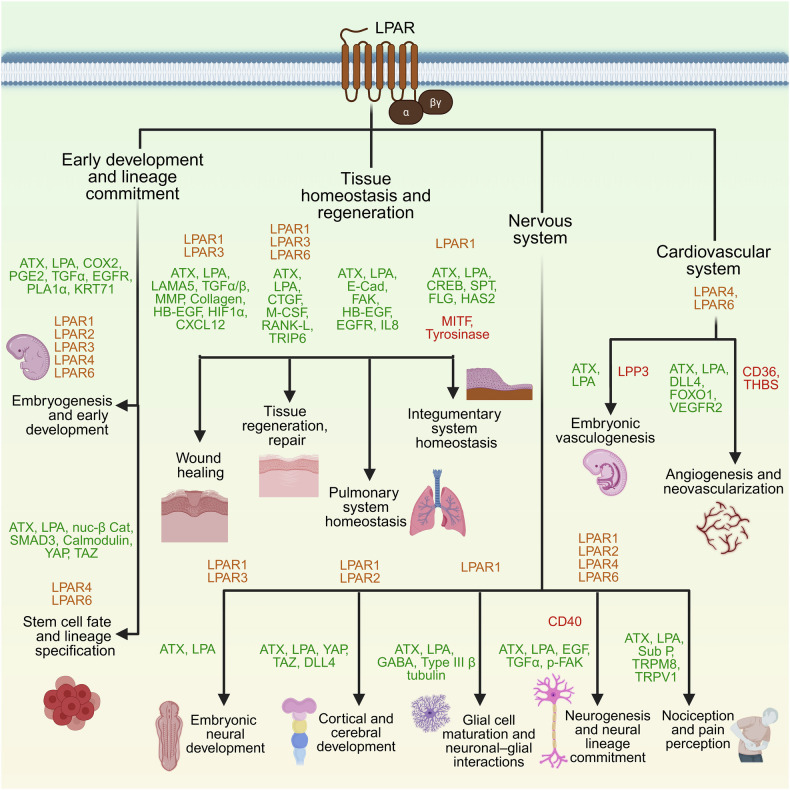
LPA signaling in normal physiology. The figure illustrates the role of LPA-LPAR signaling in normal human physiology, including embryonic development and lineage commitment; tissue homeostasis and regeneration; and the development and function of the nervous and cardiovascular systems. LPA receptors (LPARs) involved in each functional pathway are shown in orange text, while green and red text denote upregulated and downregulated signaling mediators, respectively. Expanded abbreviations representing the signaling mediators are available in the manuscript. The figure was created via BioRender.com under an academic license

LPA signaling extends beyond intracellular cascades to regulate fundamental physiological functions, from embryogenesis to tissue repair and immune modulation (Fig. 2). This section summarizes key roles of the ATX–LPA–LPAR–LPP axis across development and homeostasis. As discussed below, LPA signaling plays a critical role across different physiological contexts, beginning with its essential roles in development and extending to its regulation of adult tissue homeostasis and repair.

### LPA signaling in development and lineage commitment

LPA signaling contributes to early reproductive and embryonic events. LPAR3 is required for embryo implantation through the regulation of COX-2, PGE2 and PGI-2. Its deletion leads to delayed implantation, abnormal embryo spacing, and reduced litter sizes in female mice.^81,82^ In males, combined deletion of LPAR1/2/3 impairs germ cell survival and increases the risk of azoospermia.^82^

The LPA-LPAR axis is also involved in diverse developmental processes, including angiogenesis, osteogenesis, and follicular morphogenesis.^83^ In the retina, LPAR4/6 double knockout mice display disrupted retinal tip cell formation, which adversely affects retinal development.^1^ LPAR4 deficiency and LPAR1 activation enhance osteoblast maturation and trabecular bone formation.^84^ Furthermore, LPAR6 and P2Y5 receptors have been shown to regulate hair follicle morphogenesis and maturation via the TACE–TGFα–EGFR signaling pathway and keratin (KRT71) expression.^85–87^

In stem cells, LPA governs lineage specification. It promotes mesoderm differentiation in human iPSCs via β-catenin and neural differentiation during cortical development.^11^ LPA also promotes the pluripotency of stem cells via the YAP-TAZ signaling pathway.^88^ These dual roles reflect the capacity of LPA to modulate context-dependent developmental outcomes.

### LPA signaling in tissue homeostasis and regeneration

In addition to its developmental role, LPA performs critical regulatory functions in adult tissues by maintaining structural integrity and promoting repair. Through tightly regulated signaling networks, LPA modulates wound healing, bone remodeling, epithelial barrier maintenance, and skin physiology as follows.

#### Wound healing

LPA accelerates wound closure by promoting epithelial proliferation, migration, and extracellular matrix (ECM) remodeling. Upon injury, platelet-derived ATX elevates local LPA levels, which induce Rho-dependent laminin-5 expression and activate the TGF-α/β pathway to drive keratinocyte proliferation and differentiation.^89^ LPA also enhances immune cell infiltration, ECM thickening, and matrix remodeling via matrix metalloproteinases (MMPs).^90–94^ In corneal injury, LPA triggers HB-EGF shedding, which activates EGFR–ERK–Akt signaling to promote epithelial repair.^95^ In muscle injury, LPA–LPAR1/3 signaling activates satellite cells, supporting their proliferation and differentiation into myotubes.^96,97^ In vascular injury, this axis upregulates HIF-1α and C-X-C motif chemokine ligand-12 (CXCL-12), recruiting vascular smooth muscle progenitors and facilitating neointima formation during postinjury recovery.^98^

#### Tissue repair and regeneration

LPA supports tissue regeneration by modulating osteoblast, osteoclast, and osteocyte activities. It promotes osteoblast differentiation from mesenchymal stem cells via LPAR1 and enhances proliferation through Gαi–PI3K and Gαi–PKC signaling.^1,99,100^ Osteoblast chemotaxis is mediated by LPAR1–Gαi–Ca²⁺–ERK1/2, whereas maturation involves the LPAR3-Rho and LPAR1-Connective Tissue Growth Factor (CTGF)/Cellular Communication Network Factor 2 (CCN2) pathways.^101–103^ LPA-induced blebbing and ECM remodeling further contribute to structural repair.^104^

During bone injury, osteoblast- or platelet-derived LPA induces dendritic process extension in osteocytes, facilitating intercellular signaling during remodeling.^105^ LPA also enhances osteoclastogenesis and bone resorption via LPAR1/3 in coordination with M-CSF and RANK-L and through LPAR1-Gαi and LPAR2-c-Src-Thyroid Hormone Receptor Interacting Protein 6 (TRIP6) signaling.^106–108^

#### Pulmonary system homeostasis

LPA plays a critical role in maintaining pulmonary homeostasis by regulating epithelial barrier integrity and airway immune responses. LPA signaling supports the endothelial adherens junctions that preserve the vascular barrier of the lungs.^109^ Through the LPA–LPAR–Gαi axis, it activates protein kinase δ/ζ (PKCδ/ζ), which induces E-cadherin expression and FAK–cortactin signaling to strengthen adherens junctions and enhance transepithelial resistance.^110^ LPA also participates in the pulmonary inflammatory response, particularly in the context of allergen exposure. In bronchial epithelial cells, LPA-mediated signaling partially accounts for the inflammation triggered by airborne irritants.^111^ In response to allergens or injury, LPA stimulates fibroblast-mediated HB-EGF shedding, which transactivates EGFR on bronchial epithelial cells. This activates ERK1/2 signaling and promotes IL-8 production, facilitating neutrophil recruitment and airway immune defense.^112,113^

#### Integumentary system homeostasis

LPA regulates skin homeostasis by maintaining barrier integrity, hydration, and pigmentation. Through the LPA–LPAR1–PI3K–ERK–CREB pathway, it induces hyaluronic acid synthase 2 in dermal fibroblasts, increasing hyaluronic acid production and ECM hydration.^114^ LPA is essential for the maintenance of skin barrier function and the differentiation of keratinocytes to maintain homeostasis. LPA upregulates the expression of filaggrin, a protein involved in the skin barrier and hydration functions, through the LPAR1 and LPAR5 receptors.^115^ Mechanistically, LPA activates the RHO-ROCK-SRF signaling pathway, which is essential for the expression of filaggrin. In addition, LPA attenuates melanogenesis by suppressing microphthalmia-associated transcription factor (MITF) and tyrosinase activity, contributing to pigmentation balance.^116^ Collectively, these findings establish LPA as a pivotal regulator of tissue development, homeostasis, and regenerative processes across multiple organ systems.

### LPA Signaling in nervous system development and function

LPA signaling has profound effects on the nervous system. From shaping early neural architecture to regulating glial activity, vascular stability, and sensory processing, LPA functions as a versatile modulator of both central nervous system (CNS) and peripheral nervous system (PNS) biology as follows.

#### Embryonic neural development

LPA signaling is essential for embryonic brain formation, coordinating neural tube closure, vascular patterning, and oligodendrocyte differentiation. ATX, the primary LPA-producing enzyme, is indispensable for CNS development, as ATX-null mice exhibit embryonic lethality due to severe neural and vascular defects.^117^

These defects include avascular yolk sacs, impaired endothelial differentiation, midbrain–hindbrain boundary disruption, and abnormal forebrain expansion. Mechanistically, these effects are mediated by the ATX–LPA–LPAR–Gα12/13–Rho–ROCK pathway, which regulates neurovascular morphogenesis and progenitor cell behavior.^118^

#### Cortical and cerebral development

The LPA-LPAR1/2 axis plays a role in promoting cerebral cortex development by attenuating apoptosis, enhancing terminal mitotic signals in neural progenitor cells, and reducing their maturation during embryonic development.^119^ LPAR1-deficient mice exhibit disrupted olfactory bulb and cortical development, vascular defects, and craniofacial abnormalities, including hemorrhage, nasal hypoplasia, interocular widening, and embryonic lethality.^120^ Similarly, LPAR2-deficient mice display an increased incidence of frontal lobe hematomas, underscoring their role in brain vascular integrity.^3^ In addition, LPA–LPAR4/6 signaling through the Gα12/13-Rho-ROCK pathway activates YAP and transcriptional coactivator with PDZ-binding motif (TAZ) transcription factor, which drives delta-like ligand 4 (DLL4) expression via the β-catenin and Notch intracellular domain (NICD) pathways, contributing to CNS angiogenesis during development.^14^

#### Neurogenesis and neural lineage commitment

LPA promotes neurogenesis by enhancing the proliferation and differentiation of neural progenitor stem cells (NPSCs) and neuroblasts into cholinergic and microtubule-associated protein 2 (MAP2)-positive neurons.^24^ This is facilitated by LPAR1-Gαi/o- signaling, as evidenced by a reduction in CD140a+ve oligodendrocyte precursors and an increase in Type III β-tubulin+ve immature neurons.^121^ In addition, LPA promotes cortical neurogenesis by inducing electrical impulses in cortical neuroblasts via γ-aminobutyric acid (GABA) signaling.^122^ The role of LPA as a proneurogenic factor is further supported by its ability to induce Rho–ROCK-mediated morphological changes in NPSCs that facilitate neuronal differentiation.^11^

#### Glial cell maturation and neuronal–glial interactions

LPA also regulates the differentiation and function of nonneuronal glial cells, including astrocytes, Schwann cells, satellite cells, ependymal cells, oligodendrocytes, and microglia. In astrocytes, LPA facilitates neuronal-astrocyte interaction that mediates neuronal commitment, cortical neuron differentiation and neurite outgrowth through activation of LPA-LPAR1/2-EGF-TGFα axis.^123–125^ LPAR1 also promotes embryonic Schwann cell proliferation, facilitating sciatic nerve myelination.^126^ In oligodendrocyte precursor cells (OPCs), LPA supports myelination via the ATX–LPAR1 signaling axis.^127^ Furthermore, LPA mediates oligodendrocyte maturation in the central nervous system by reducing their interaction with ECM components through a reduction in FAK phosphorylation through the ATX-LPA-LPAR-Gαi axis.^128–130^ However, context-specific antidifferentiation effects of LPA have also been reported in both OPCs and NPSCs.^131,132^

#### Nociception and pain perception

LPA–LPAR1 signaling has also been implicated in nociception and pain perception. The LPA-LPAR1 axis activates transient receptor potential cation channels (transient receptor potential melastatin 8 (TRPM8) and transient receptor potential vanilloid 1 (TRPV1)) on C-fiber nociceptors, triggering substance P release and chemical nociception.^133^ LPA-LPAR signaling has also been implicated in pain associated with fibromyalgia. In mouse models of fibromyalgia, genetic deletion of LPAR1 or LPAR3 significantly attenuates mechanical and stress-induced pain, highlighting the role of LPA in pain amplification.^134^

### LPA signaling in cardiovascular system development and function

The cardiovascular system, which comprises endothelial cells, blood vessels (arteries, veins, capillaries), the heart, and circulating blood components, is profoundly regulated by LPA signaling. Emerging evidence has demonstrated that LPA modulates vascular development, endothelial cell behavior, and angiogenic remodeling through both direct receptor-mediated signaling and paracrine modulation.

#### Embryonic vasculogenesis

LPA signaling is essential for embryonic vascular development. ATX deficiency leads to embryonic lethality due to failure of vasculogenesis, as shown in zebrafish models.^135^ Similarly, deletion of LPAR4 or LPAR6 impairs vascular formation and results in embryonic lethality.^14,136,137^

However, proper vessel development requires not only the presence of LPA but also tightly controlled spatial gradients. Loss of the LPA-degrading enzyme LPP3 results in elevated LPA levels but causes disorganized vasculature due to loss of spatiotemporal signaling control.^138,139^

#### Angiogenesis and neovascularization

LPA regulates developmental angiogenesis primarily through the activation of the Rho-ROCK signaling pathway. In endothelial cells, LPA-LPAR4/6 signaling through the Gα12/13-Rho-ROCK pathway activates the transcription factors YAP and TAZ, promoting DLL4 expression via β-catenin and NICD, thereby orchestrating CNS angiogenesis and vascular sprout.^14^

The vascular defects observed in LPAR4 and LPAR6 double-knockout mice, including impaired vascular sprouting, defective tip cell formation, disrupted branching morphogenesis, and embryonic lethality, directly implicate LPA-LPAR signaling in promoting endothelial cell migration, proliferation, and network formation during vessel sprouting.^14^ Furthermore, the dilation of lymphatic vessels and defective mural cell recruitment observed in LPAR4- and Gα13-knockout embryos further highlight the role of LPA-LPAR4-Gα13 signaling in stabilizing nascent vessels through mural cell associations.^136^

At the cellular level, LPA activates PKD1–FOXO1 in endothelial cells, downregulating antiangiogenic factors such as CD36 and thrombospondins while enhancing VEGFR2 responsiveness and promoting survival and tube formation.^140,141^ In addition to promoting vessel sprouting, LPA safeguards the integrity of the emerging vasculature by strengthening endothelial barriers, a crucial step for the maturation and functional integration of newly formed vessels. This stabilizing function is exemplified by platelet-derived LPA, which lowers vascular endothelial permeability and enhances endothelial barrier integrity.^142^

Across organ systems, LPA–LPAR signaling coordinates critical physiological processes, from embryonic development and stem cell lineage specification to tissue regeneration and vascular stabilization. Through tightly regulated spatiotemporal gradients and receptor-specific signaling cascades, LPA modulates epithelial integrity, neurogenesis, the immune response, myelination, and angiogenesis.

## LPA signaling in disease pathogenesis

While LPA signaling is intricately linked with vital processes in normal physiology, its dysregulation profoundly reshapes the cellular microenvironment to drive disease pathogenesis. Aberrant activation of the LPA-LPAR axis fuels chronic inflammation, fibrotic remodeling, tumor progression, metabolic dysfunction, neurodegeneration, and vascular instability. This section examines how the pathological rewiring of LPA signaling contributes to disease initiation, progression, and therapeutic resistance across diverse organ systems.

### LPA signaling in cardiovascular diseases

**Fig. 3:**
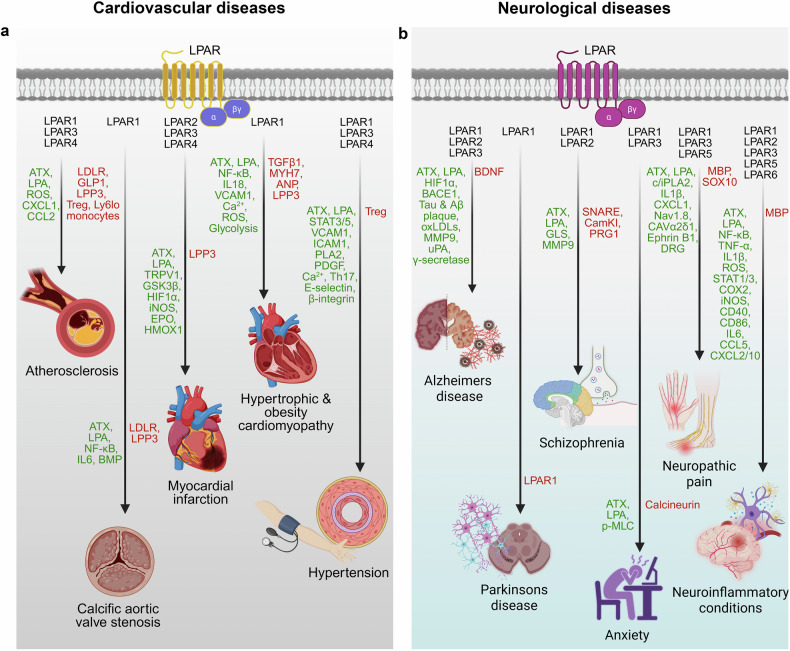
LPA signaling in the pathobiology of cardiovascular and neurological diseases. The figure illustrates the role of LPA-LPAR signaling in the pathobiology of **a** cardiovascular diseases, including atherosclerosis, calcific aortic valve stenosis, myocardial infarction, hypertrophic cardiomyopathy, obesity-associated cardiomyopathy and hypertension, as well as **b** neurological diseases, including Alzheimer’s disease, Parkinson’s disease, schizophrenia, anxiety, neuropathic pain and other neuroinflammatory conditions. LPA receptors involved in each functional pathway are represented in black text. The upregulated and downregulated signaling mediators are represented in green and red text, respectively. Expanded abbreviations for the downstream mediators are provided in the manuscript. Figure created via BioRender.com under an academic license

The cardiovascular system is profoundly influenced by LPA signaling, which regulates vascular integrity, inflammatory responses, and tissue remodeling. While low levels of LPA promote vascular repair and cardiac regeneration, aberrant activation of LPA pathways contributes to the pathogenesis of several major cardiovascular diseases. Elevated LPA levels and dysregulated LPAR signaling have been implicated in atherosclerosis, calcific aortic valve disease (CAVD), hypertrophic and obesity-associated cardiomyopathies, myocardial infarction (MI), and hypertension (Fig. 3a). In this section, we delineate the mechanism by which LPA orchestrates cardiovascular dysfunction, highlighting emerging therapeutic insights.

#### LPA and atherosclerosis

Atherosclerosis, a leading cause of MI, is characterized by fibrous‒fatty lesion formation within arterial walls and remains a major contributor to cardiovascular morbidity and mortality.^143^ LPA acts as a potent proinflammatory mediator in atherosclerosis.^2^ Elevated levels of LPA, specifically unsaturated long chain acyl-LPA species, have been detected within atherosclerotic plaques and are correlated with increased expression of LPARs and dysregulated levels of enzymes that regulate LPA metabolism.^144^ In pathological states such as hyperlipidemia, LPA shifts from albumin-bound to lipoprotein (a)- or low-density lipoprotein (LDL)-associated forms, concentrating near the endothelium and enhancing local pro-atherogenic signaling.^145,146^ In Ldlr⁻/⁻ mice, which lack LDL clearance, an ~20-fold increase in LPA, especially LDL-bound unsaturated species, is observed in advanced lesions, implicating LPA in plaque expansion.^147–150^ Dietary factors further exacerbate this process. A Western diet further exacerbates this effect by increasing the level of ROS, which induces Enpp2 (ATX) expression in enterocytes and aortic endothelial cells, increasing systemic and local LPA levels.^151^

Endothelial cells of the aorta also show increased *Enpp2* expression, leading to increased ATX accumulation on the endothelial surface. This promotes local LPA synthesis and secretion, increasing plasma LPA levels. In turn, this induces CXCL1 expression, facilitating monocyte adhesion and macrophage infiltration, further exacerbating atherosclerosis.^152^ In support of these findings, perivascular administration of LPA in vivo enhances intraplaque hemorrhage by recruiting inflammatory mast cells into atherosclerotic plaques in carotid arteries in a mouse model.^147^

LPA promotes atherosclerosis primarily through LPAR1 and LPAR3, which induce CXCL1 and CCL2 to recruit neutrophils and monocytes in atheromas^153^72. LPAR4 has been linked to increased endothelial permeability, lipid accumulation, lymphocyte migration, and macrophage phenotype switching.^153,154^ LPA also drives foam cell formation, furthering atheroma progression.^155^ The pan-LPAR antagonist Ki16425 reduces disease burden by increasing the numbers of anti-inflammatory T regulatory (Treg) cells and Ly6C-low monocytes.^156^

LPA also contributes to glucose intolerance and insulin resistance by suppressing GLP-1 secretion, an effect reversible by LPAR1/3 antagonists.^157,158^ Given that GLP-1 receptor agonists reduce type 2 diabetes and cardiovascular risk, this link adds metabolic relevance to the pathogenic role of LPA.^159^

Genetic studies have identified *PLPP3*, which encodes LPP3, as a coronary artery disease (CAD) risk locus.^160,161^ LPP3 attenuates LPA signaling by hydrolyzing LPA to MAG, suppressing vascular inflammation, supporting smooth muscle cell (SMC) differentiation, and maintaining endothelial integrity.^138,162,163^ While these findings support the pathogenic role of LPA in atherosclerosis, in the case of abdominal aortic aneurysm, which is yet another complication of atherosclerosis, the deletion of LPP3 and the consequent increase in LPA levels protected mice from angiotensin-II-mediated aneurism. These findings suggest that LPA plays a protective role against abdominal aortic aneurysm.^164^ However, in abdominal aortic aneurysm models, LPP3 deletion and consequent LPA elevation paradoxically protect against aneurysm formation, suggesting that context-specific LPA functions are dependent on vascular SMC origin.

#### LPA and calcific aortic valve disease

CAVD is characterized by progressive thickening and calcification of the aortic valve due to osteogenic transformation of valve interstitial cells, eventually leading to aortic stenosis and obstruction of blood flow.^165^ Clinical studies have shown that patients with CAVD exhibit elevated levels of ATX and reduced levels of LPP3, with the resulting dysregulation of LPA metabolism.^165,166^ Epigenetic silencing of *LPP3* via DNA methylation at its enhancer contributes to local LPA accumulation and disease progression.^166^ In addition, ATX, which is transported by lipoprotein(a) or platelets, accumulates in valve interstitial cells where it generates LPA, promoting calcification.^54,167–169^ The role of LPA in CAVD is further substantiated by clinical observations that elevated plasma LPA levels are positively correlated with CAVD severity and hemodynamic worsening.^170^

Mechanistically, LPA drives CAVD progression primarily through activation of the NF-κB-interleukin-6 (IL-6)-bone morphogenetic protein (BMP) pathway.^167,168^ LPAR1-mediated signaling via Rho and NF-κB also contributes to disease progression.^54^ Experimental studies have confirmed that LPA administration accelerates aortic valve calcification and accelerates the onset of CAVD in LDLR–/–/ApoB100/100/IGFII mice.^168^

#### LPA and hypertrophic cardiomyopathy

HCM, a common inherited cardiac disorder, is characterized by myocardial thickening and fibrosis, increasing the risk of arrhythmias, stroke, and heart failure.^171^ LPA–LPAR1 signaling, broadly implicated in fibrotic diseases, contributes significantly to cardiac fibrosis.^171^ In the heart, LPAR1 is enriched in left ventricular fibroblasts, endothelial cells, and lymphatic endothelial cells (LECs) but is minimally expressed in cardiomyocytes.^171^ LPAR1 deletion in mouse models reduces LEC populations, disrupts fibroblast transitions, and attenuates ventricular wall thickening and fibrosis, ameliorating HCM features.^171^

At the mechanistic level, LPA-LPAR-Gaq–PLC signaling in LECs increases intracellular Ca²⁺ levels, elevates intracellular Ca²⁺ in LECs, and activates calpain and NF-κB, which induces IL-18–mediated VCAM1 expression and vascular inflammation. Simultaneously, LPA-activated mitogen-activated protein kinase kinase kinase 1 (MEKK1) signaling suppresses transforming growth factor-β1 (TGF-β1) expression, impairing Treg stability and promoting the proinflammatory milieu leading to HCM pathogenesis.^172,173^

In support of these findings, in vivo studies using mouse stroke models have demonstrated that ATX knockout reduces LPA levels, improves vascular permeability, enhances cerebral blood flow, and decreases infarct volume, collectively reducing HCM-associated vascular dysfunction.^174^

#### LPA and obesity cardiomyopathy

LPA signaling also contributes to obesity-related cardiac dysfunction, linking metabolic stress to pathological myocardial remodeling. Obesity cardiomyopathy is driven by metabolic and inflammatory stressors, including increased fatty acid uptake, lipid overaccumulation, impaired mitochondrial function, insulin resistance, endoplasmic reticulum (ER) stress, and chronic inflammation, resulting in left ventricular hypertrophy, diastolic dysfunction, cardiac fibrosis, and eventual heart failure.^175,176^

ATX/LPA plays a central role in obesity-related cardiomyopathy, as evidenced by ATX knockout and ATX inhibitor PF-8380-based studies. Cardiomyocytes obtained from global heterozygous ATX-deficient mice presented normal insulin-activated phospho-Akt levels and contractility upon consumption of a high-fat/high-sucrose diet.^79^ Pharmacologic ATX inhibition with PF-8380 lowers serum LPA levels, reduces ventricular wall thickening, increases ANP and β-MHC expression, limits inflammation, and improves cardiac function.^177^ Adipose-specific ATX knockdown preserves mitochondrial function, enhances citrate synthase activity, and reduces myocardial steatosis.^178^ Thus, ATX/LPA signaling has emerged as a critical driver of obesity-induced cardiomyopathy. At the receptor level, LPAR1 knockdown exacerbates LPA-induced cardiomyocyte hypertrophy, whereas LPAR3 knockdown attenuates hypertrophy, suggesting that different LPARs play different roles in regulating cardiac remodeling hypertrophy.^179^

In addition, LPP3 further regulates cardiac LPA homeostasis. Cardiomyocyte-specific LPP3 deletion enhances ERK and Rho activation, inducing ventricular tachycardia, reduced contractility, and hypertrophy.^180^ LPP3 loss also disrupts mitochondrial dynamics by increasing superoxide production, impairing oxygen consumption and ATP synthesis, and shifting metabolism toward glycolysis, collectively driving bioenergetic failure and myocardial remodeling in obese individuals.^181–183^

#### LPA and myocardial infarction

MI results from interrupted coronary blood flow, leading to ischemic injury, cardiomyocyte necrosis, and heart failure, driven by disrupted energy metabolism, calcium handling, mitochondrial dysfunction, and cell death pathways, including necrosis, ferroptosis, and autophagy.^184,185^

LPA signaling exerts context-specific effects during MI. LPAR2 expression in cardiac endothelial cells is upregulated early post-MI, promoting angiogenesis, reducing fibrosis, and preserving function.^186^ In cardiomyocytes, LPA activates the PI3K-Akt, Hippo-YAP, BMP-Smad1/5, and MAPKK-ERK pathways, with LPAR3 critically regulating ERK-driven repair.^187^ High LPAR3 expression is correlated with improved regeneration, whereas LPAR4 is associated with worsened outcomes^187,188^

Despite the reparative roles of LPAR2/3, elevated systemic LPA after MI exacerbates injury. Increased serum LPA levels are correlated with inflammatory infiltration, systolic dysfunction, hypertrophy, and autophagy inhibition via LPAR3–Akt–mTOR signaling.^189,190^ Under hypoxic conditions during MI, the stabilization of HIF1α typically promotes the expression of cardioprotective genes, including erythropoietin, heme oxygenase-1, and inducible nitric oxide synthase (iNOS), and shifts cellular metabolism from oxidative phosphorylation to anaerobic glycolysis to limit ischemic injury.^191,192^ LPA-driven HIF1α stabilization, rather than promoting protective glycolytic adaptation, enhances monocyte/macrophage activation and fibrosis, worsening ischemic injury.^193^

ATX inhibition via PF-8380 or HA130 reduces inflammation, augments angiogenesis, improves recovery, and decreases cardiac sympathetic activity post-MI^.^^190,194^ In contrast, LPP3 suppression enhances LPA levels and promotes systemic inflammation by expanding bone marrow-derived myeloid progenitors and increasing proinflammatory Ly6C-high monocyte infiltration.^190,195,196^

#### LPA and hypertension

LPA signaling contributes to hypertensive disorders, particularly pulmonary hypertension. In hypoxia-induced pulmonary arterial hypertension, LPA activates RhoA–ROCK and disrupts the Th17/Treg balance via STAT3/STAT5 phosphorylation, promoting immune dysregulation.^197^ These effects drive vascular remodeling through immune cell recruitment. Furthermore, ATX‒LPA signaling via LPAR4‒Gα12/13 activation has also been implicated in the elevation of systemic blood pressure.^1^

In systemic hypertension, increased platelet activation and phospholipase A2 activity increase LPA production, which promotes platelet aggregation and PDGF release, further amplifying LPA generation and vascular smooth muscle cell (VSMC) proliferation. LPA also increases intracellular calcium levels in VSMCs, enhancing vascular tone and contributing to elevated blood pressure.^198^

Collectively, these findings underscore LPA’s central role in cardiovascular pathophysiology, influencing vascular integrity, cardiac remodeling, and systemic hypertension.

### LPA signaling in neurodevelopmental, neurodegenerative, and psychiatric disorders

LPA signaling plays a critical role in the nervous system, influencing processes from early neurodevelopment to the progression of neurodegenerative and psychiatric disorders. This section explores the roles of LPA, its receptors, and associated signaling pathways in conditions such as Alzheimer’s disease (AD), Parkinson’s disease (PD), schizophrenia, neuropathic pain, anxiety, and neuroinflammatory syndromes (Fig. 3b).

#### LPA and Alzheimer’s disease

AD is characterized by β-amyloid (Aβ) plaque accumulation and tau hyperphosphorylation, leading to synaptic dysfunction and cognitive decline.^199,200^ Elevated ATX activity in AD increases CNS LPA levels, particularly in the frontal cortex, where LPA–LPAR signaling activates the Gi–PKCδ–MEK–ERK–p90RSK–CREB axis, increasing BACE1 and promoting Aβ generation.^201,202^ In addition to amyloidogenesis, LPA also drives tau hyperphosphorylation via Gα12/13–RhoA–GSK3β, which target the Thr245 and Ser409 residues,^203–205^ with additional contributions from PKC and p38MAPK that contribute to neurite retraction.^206^ LPA also induces neuronal apoptosis, impairs astrocytic glutamate uptake, and disrupts axonal structure and blood–brain barrier integrity through Rho–ROCK–MMP9/uPA signaling.^207,208^

Furthermore, several AD risk factors, including traumatic brain injury (TBI), metabolic syndrome, and chronic hypoperfusion, are associated with dysregulated LPA signaling. TBI increases LPAR1–3 expression and LPA levels, exacerbating Aβ deposition, tau pathology, and neuroinflammation.^209,210^ Metabolic syndrome promotes oxidative stress and barrier disruption via ATX–LPA signaling.^211–213^ Under conditions of cerebral hypoperfusion, LPA signaling enhances HIF-1α stabilization, which in turn increases BACE1 expression and γ-secretase activity, thereby accelerating Aβ production and contributing to AD progression.^214–216^

#### LPA and Parkinson’s disease

Parkinson’s disease (PD) is a progressive neurodegenerative disorder caused by the selective degeneration of dopaminergic (DA) neurons in the substantia nigra, resulting in dopamine depletion in the striatum.^217^ LPA signaling has been implicated both in the differentiation of DA neurons from mesenchymal stem cells and in the degeneration of DA neurons. In addition, LPAR1 expression is reduced in the substantia nigra but preserved in the striatum in PD models, suggesting a region-specific role.^218^ Although direct studies remain limited, emerging evidence supports a contributory role for LPA dysregulation in PD pathogenesis.

#### LPA and schizophrenia

Schizophrenia is a chronic psychiatric disorder characterized by cognitive and behavioral abnormalities arising from genetic and neurodevelopmental disruptions.^219^ In vivo studies have correlated the role of LPAR1 deficiency with schizophrenia-related abnormalities, including prepulse inhibition and dysregulation of presynaptic SNARE and hippocampal CaMKII complexes.^220^ LPA–LPAR1 signaling regulates neurotransmitter release (e.g., tyrosine, glutamate, and GABA), and its disruption induces schizophrenia-like features in mice.^221^ Notably, prenatal LPA exposure induces schizophrenia-like symptoms, which are attenuated by LPAR1 knockout or inhibition.^222^

At the molecular level, the LPA–LPAR1 axis modulates glutamatergic transmission and neuroplasticity, further linking it to schizophrenia.^223^ Moreover, astrocyte-derived ATX plays a role in regulating glutamatergic synaptic transmission, and its inhibition has been shown to attenuate schizophrenia-related hyperexcitability syndrome.^224^ In addition to LPAR1, schizophrenia has also been linked to LPA–LPAR2 signaling, which regulates the expression of plasticity-related gene 1 (PRG-1), a key modulator of synaptic LPA signaling and cortical hyperexcitability associated with schizophrenia syndrome.^224^

#### LPA and neuropathic pain

Neuropathic pain, a chronic condition affecting the somatosensory system, often arises independently of nociceptive transduction.^225,226^ LPA-activated LPAR1/3 and associated signaling pathways play significant roles in inducing chronic neuropathic pain.^1,227^ Among LPA species, 16:0, 16:1, and particularly 18:1 have been correlated with pain intensity in clinical patient samples. Specifically, LPA 18:1 has been correlated with chronic neuropathic pain through LPAR1- and LPAR3-mediated signaling for microglial activation pain.^1^

LPA also induces C-fiber retraction, A-fiber demyelination, and dorsal root fiber sprouting, contributing to allodynia and synaptic reorganization pain.^227^ Mechanistically, LPA–LPAR1 activates the Gα12/13–RhoA–ROCK–PKCγ pathway, promoting dorsal root demyelination and upregulating Ephrin B1 and CAVα2δ1 expression during early nerve injury.^33,228–230^ Microglia-derived LPA further activates astrocytes to secrete chemokines, amplifying inflammatory signaling,^227,231^ and stimulates MAPKs^228^ and p38MAPKs for pain persistence.^232–234^

In partial sciatic nerve ligation (pSNL) models, peripheral nerve stimulation via NMDA and substance P activates cytosolic iPLA2, generating LPC, which ATX converts to LPA. This signals through LPAR3 to induce IL-1β via ERK1/2 phosphorylation, with downstream activation of neurons and CXCL1 secretion from astrocyte lesions.^227–229,232,235^ Furthermore, LPA–LPAR1/3 signaling establishes a positive feedback loop that enhances LPA synthesis and promotes the activation of macrophages and microglia, partly through LPAR5–PKD signaling, to induce neuropathic pain. In osteoarthritis models, LPA exacerbates mechanical allodynia, particularly in females, largely via the voltage-gated sodium channel Nav1.8.^236^

#### LPA and anxiety

Anxiety and depression are prevalent psychiatric disorders that are often accompanied by impaired neuromuscular and mood regulation.^237^ Reduced ATX levels in the serum and CSF of affected patients have been linked to impaired glial development and mood instability.^238,239^ LPA-LPAR1 signaling plays a central role in anxiety regulation,^240,241^ with LPAR1 deficiency leading to heightened anxiety-like behaviors, diminished neuromuscular strength, and altered analgesia.^220,223^ LPAR3 deletion has also been associated with increased anxiety symptoms.^242^

At the synaptic level, LPA–LPAR1 modulates glutamatergic and GABAergic transmission via the Gαi/o–PLC–MLCK and Gα12/13–RhoA–ROCK–calcineurin pathways, respectively. Disruption of these pathways impairs developmental and behavioral regulation, contributing to anxiety and mood disorders.^243^

#### LPA and neuroinflammatory disorders

LPA–LPAR1 signaling drives neuroinflammation by activating NF-κB, promoting proinflammatory cytokine release, and inducing microglial transformation following ischemic injury.^39,244^ This pathway is also implicated in intracerebral hemorrhage, spinal cord demyelination, and sepsis-induced microglial TNF-α release.^244–247^ In astrocytes, LPA–LPAR1 activates ERK1/2 and stimulates IL-1β release, aggravating cerebral ischemia and inflammatory damage.^235,248^

LPAR-mediated neuroinflammatory responses are context specific: LPAR1 activates NF-κB signaling to induce proinflammatory cytokine release and microglial activation^39,244^; LPAR2 promotes oligodendrocyte death following spinal injury^249^; LPAR3 enhances reactive oxygen species (ROS) production in microglia^250^; and LPAR5 drives M1 microglial polarization via MAPK–NF-κB–STAT1/3 signaling, worsening ischemic brain injury.^218,251–254^

### LPA Signaling in metabolic and endocrine diseases

**Fig. 4:**
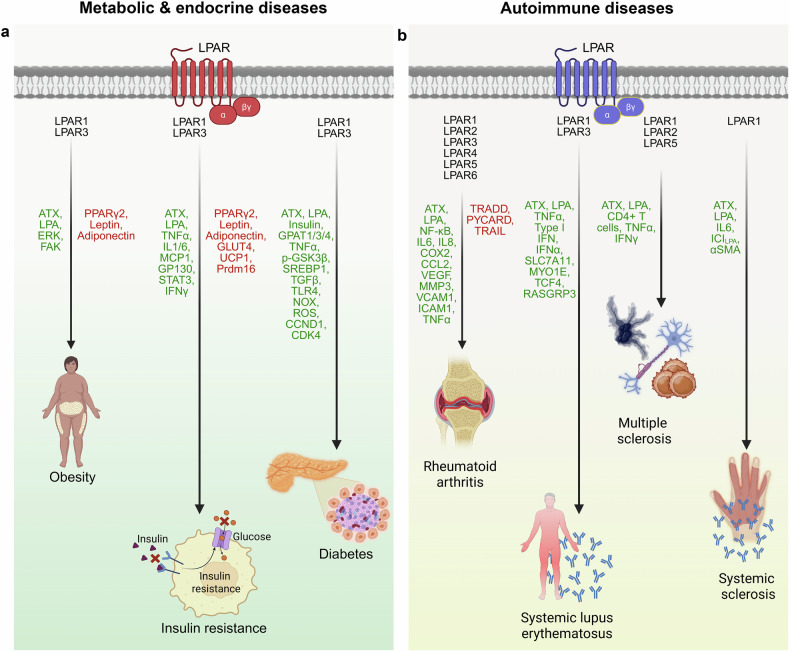
LPA signaling in the pathobiology of metabolic/endocrine and autoimmune diseases. The depicts the role of LPA-LPAR signaling in the pathobiology of **a** metabolic and endocrine diseases such as obesity, insulin resistance and diabetes, and **b** autoimmune diseases such as rheumatoid arthritis, systemic lupus erythematosus, multiple sclerosis and systemic sclerosis. LPA receptors involved in each functional pathway are represented in black text. Upregulated and downregulated signaling molecules are denoted by green and red text, respectively. Expanded abbreviations are detailed in the manuscript. The figure was created via BioRender.com under an academic license

LPA-mediated signaling regulates numerous metabolic and endocrine diseases, including obesity, diabetes, and insulin resistance (Fig. 4a).

#### LPA and obesity

Obesity is a major risk factor for diabetes, cardiovascular disease, and cancer. The ATX–LPA axis is central to adiposity regulation, acting through both systemic and adipose-localized mechanisms. High-fat and obesogenic diets increase the levels of circulating LPA, especially unsaturated species, in various mouse models (e.g., FVB, C57BL/6 J, and LDLR⁻/⁻), with plasma LPA levels correlated with body mass index (BMI).^79,150,255–257^ Elevated ATX expression and activity in adipose tissue, along with increased circulating ATX and LPA levels, have been observed in high-fat diet-induced obesity models.^80,255,256^ Consistently, obese individuals present increased ATX mRNA levels in visceral adipose tissue, elevated serum ATX concentrations, and increased 16:0 LPA species, all of which are correlated with BMI.^258–260^

ATX–LPA signaling regulates both adipocyte hyperplasia and hypertrophy. LPA–LPAR1 signaling promotes preadipocyte proliferation and favors the white over brown adipocyte lineage via the Ras–Raf–MEK–ERK and FAK pathways.^53^ Lpar1⁻/⁻ mice are resistant to diet-induced obesity due to altered leptin regulation.^255^ However, adipose-specific ATX knockout reduces preadipocyte numbers in fat pads.^53^ LPA-LPAR1 signaling promotes preadipocyte proliferation through the Ras-Raf-MEK-ERK pathway as well as FAK activation.^261^ ATX expression increases during adipocyte differentiation, and its loss impairs adipogenesis by downregulating the expression of adipogenic markers.^53,79^

In contrast to these adipogenic roles, some studies have reported that LPA inhibits preadipocyte proliferation and differentiation, primarily through LPA-LPAR1-Rho-ROCK signaling, leading to the downregulation of PPARγ2 and associated adipogenic proteins.^262^ Adipose-specific ATX deletion has been shown to increase adipose tissue mass by increasing PPARγ2, leptin, and adiponectin expression, promoting adiposity.^53,79^ Furthermore, global heterozygous ATX knockout and adipose-specific ATX-deficient C57BL/6 mice were protected from diet-induced obesity, whereas adipose-specific ATX overexpression promoted adiposity and obesity obesity.^255^ Pharmacological inhibition of LPAR1/3 with Ki16425 also promoted fat mass and expansion of white adipocytes in high-fat diet–fed mice.^259^ ATX inhibition also favored brown preadipocyte differentiation over white preadipocyte differentiation.^262^ In addition, clinical studies have reported reduced ATX levels in adipose tissue and circulation in certain cohorts of obese humans, showing an inverse correlation with BMI.^53^ Although the reasons for these seemingly contradictory findings remain unclear, they may reflect differences in genetic background, tissue microenvironment, or experimental models used across studies.

#### LPA and insulin resistance

The LPA signaling axis plays a critical role in glucose metabolism and insulin sensitivity. Elevated serum ATX levels are correlated with impaired glucose homeostasis in obese and aging individuals.^258,260^ Similar associations have been reported between ATX expression in intra-abdominal adipose tissue and glucose intolerance in diabetic women.^263^ Experimental models support these associations. ATX knockout mice show improved insulin sensitivity on a high-fat diet, whereas exogenous LPA induces glucose intolerance, an effect reversed by the LPAR1/3 antagonist Ki16425.^53,255,264^ Chronic Ki16425 treatment enhances hepatic glycogen storage and glucose oxidation in muscle and suppresses gluconeogenesis.^264^ LPA also impairs insulin signaling by reducing AKT phosphorylation in adipocytes and attenuating glycogen synthesis via LPAR3 in hepatocytes.^265^ Furthermore, it regulates brown adipose tissue (BAT) function by downregulating UCP1, PR domain-containing 16 (Prdm16), and mitochondrial genes, reducing energy expenditure and altering the mitochondrial membrane potential, thus worsening insulin resistance.^262^

ATX-LPA signaling also negatively regulates the activity of PPARγ, a key regulator of glucose homeostasis. While TZDs improve insulin sensitivity by activating PPARγ and suppressing ATX, LPA signaling inhibits the expression of PPARγ target genes such as adiponectin, GLUT-4, and leptin.^53,79,255,263^

A major mechanism linking LPA to insulin resistance is inflammation. Obesity-associated cytokines (TNF-α, IL-1β, IL-6, MCP-1, and IFN-γ) stimulate ATX expression in adipose tissue, increasing systemic LPA levels.^53,256,263^ LPA–LPAR1/3 signaling further promotes IL-6 secretion, which activates gp130–JAK–STAT3 in adipocytes, driving ATX expression and insulin resistance.^256^ Chronic LPA signaling also contributes to adipose fibrosis via the HIF1α–TGFβ axis, promoting ECM remodeling and compounding insulin resistance. Inhibition of LPAR1/3 reduces fibrosis and improves metabolic parameters in diabetic mice.^266–268^

#### LPA and diabetes

LPA signaling has emerged as a key contributor to both type 2 diabetes (T2D), characterized by insulin resistance and impaired insulin secretion, and type 1 diabetes (T1D), an autoimmune disorder in which pancreatic β-cells are targeted. Although traditionally viewed as pathogenic in diabetes, LPA may also modulate certain disease outcomes in a context-dependent manner.

Numerous studies have demonstrated that ATX‒LPA signaling disrupts glucose homeostasis and contributes to T2D pathogenesis. Elevated ATX expression in adipose tissue and increased circulating ATX levels have been consistently reported in diabetic patients.^260,263,269^ In the db/db mouse model (leptin receptor–mutated mouse model for T2D), ATX expression was significantly upregulated in adipocytes and correlated more strongly with hyperglycemia than with hyperinsulinemia.^263^

Mechanistically, LPA promotes glucose imbalance in diabetes through multiple pathways, including dysregulation of insulin levels and activation of hepatic glycogenolysis.^270,271^ Consistent with these findings, adipose-specific ATX knockout mice exhibit improved glucose tolerance.^255^ Moreover, rosiglitazone downregulates ATX expression, supporting its role in insulin sensitization.^263^ ATX levels are also modulated by disease state and therapy. While short-term insulin therapy increases ATX activity, chronic insulin exposure enhances both ATX expression and activity.^80^ In addition, glycerol-3-phosphate acyltransferases (GPAT1/3/4), enzymes involved in LPA synthesis, are overexpressed in T2D and linked to peripheral and hepatic insulin resistance.^272^

Paradoxically, some studies suggest a protective role for LPA in glucose uptake. LPA-LPAR1/3 signaling has been shown to promote GLUT-4 translocation and enhance glucose uptake via the PI3K pathway, improving glycemic control in mice.^265,273^ These conflicting observations likely reflect differences in receptor subtype, tissue specificity, metabolic context, or timing of LPA exposure.

Among diabetes complications, diabetic nephropathy (DN) is a key manifestation marked by renal fibrosis and angiogenesis. LPA levels are elevated in the glomeruli of eNOS-deficient db/db mice and in the urine of DN patients.^274^ Antagonizing LPAR1/3 in db/db and STZ-induced T1D models reduced GSK3β phosphorylation, SREBP1 nuclear translocation, and TGF-β expression, thereby limiting fibrosis and renal injury.^275^ LPAR1 further contributes to DN through the activation of the TLR4-NF-κB and NOX–ROS inflammatory pathways.^276^

Together, these findings highlight the complex and context-dependent roles of the ATX‒LPA signaling axis in diabetes pathogenesis, influencing both glucose metabolism and the progression of diabetes-associated complications.

### LPA signaling in autoimmune diseases

LPA signaling orchestrates a range of immune-modulatory effects that contribute to the pathogenesis of multiple autoimmune diseases. Increased synthesis of LPA, altered receptor engagement and downstream signaling cascades have been implicated in the development and progression of disorders such as rheumatoid arthritis (RA), systemic lupus erythematosus (SLE), multiple sclerosis (MS), systemic sclerosis (SS), and autoimmune kidney diseases (Fig. 4b). In this section, we highlight the emerging roles of LPA, its receptors, and regulatory enzymes across these autoimmune contexts.

#### LPA and rheumatoid arthritis

Rheumatoid arthritis (RA) is a systemic autoimmune disease characterized by synovial inflammation, fibroblast-like synoviocyte (FLS) hyperproliferation, and infiltration of immune cells, leading to joint destruction.^277–279^ Elevated levels of ATX and LPA have been detected in RA synovial fluid and FLSs, particularly near sites of cartilage degradation.^280^ Synovial LPA production is driven by both resident FLSs and infiltrating immune cells and is accompanied by increased expression of LPARs, notably LPAR1–3^281,282^ and, more recently, LPAR4–6.^283^

LPA–LPAR1 signaling in FLSs activates the Gαi/o-p38/ERK–NF-κB pathways, increasing the expression of proinflammatory mediators, including IL-6, IL-8, COX-2, CCL2, VEGF, MMP3, VCAM-1, and ICAM-1.^283,284^ LPAR3 signaling via Gα12/13-Rho-p38 MAPK further enhances cytokine secretion and FLS motility.^284,285^ LPA also promotes pseudoemperipolesis, the migration of CD8 + , CD4 + , and CD19+ cells beneath the FLS layer, and bone marrow stem cell migration via LPA-LPAR1 signaling.^281,283,286^ ATX is upregulated by TNF-α, which is secreted by RA FLS, reinforcing LPA-LPAR1-mediated FLS proliferation and resistance to apoptosis.^282,287^

In vivo studies using RA mouse models, including TNF-transgenic, collagen-induced arthritis (CIA), and K/BxN models, have demonstrated that ATX ablation or pharmacologic inhibition of LPARs significantly reduces RA incidence and severity, synovial inflammation, bone erosion, and cartilage damage.^106,281,287^ These protective effects are linked to decreased osteoclast activity and enhanced osteoblast differentiation, underscoring the dual inflammatory and bone-regulatory roles of LPA signaling in RA.

#### LPA and systemic lupus erythematosus

Systemic lupus erythematosus (SLE) is a chronic autoimmune disease driven by autoantibody (e.g., anti-dsDNA, antinuclear) and immune complex deposition across multiple organs, including the kidneys, CNS, and skin.^288^

Elevated ATX levels, which are responsible for extracellular LPA generation, have been consistently observed in the serum and urine of SLE patients,^289^ particularly those with lupus nephritis, a severe renal manifestation of SLE. Transcriptomic analyses have shown upregulated expression of the ENPP2 gene (encoding ATX) in plasmacytoid dendritic cells (pDCs) from SLE patients, indicating increased intrinsic LPA-generating capacity within the immune compartment of patients.^290^

A defining feature of SLE pathogenesis is the overproduction of type I interferons by pDCs, which drive dendritic cell maturation, T-cell activation, B-cell stimulation, and autoantibody production.^291–293^ ATX-driven LPA signaling is positively correlated with type I interferon signatures in pDCs.^294^ Mechanistically, the upregulation of solute carrier family 7 member 11 (SLC7A11) leads to ENPP2 and myosin 1E (MYO1E) expression, promoting ATX-driven, LPA-dependent IFN-α and TNF-α secretion.^295^ LPA also modulates the transcription factor transcription factor 4 (TCF4), which regulates pDC differentiation and function.^296^ Gene coexpression analyses have linked ATX expression to SLE risk alleles, such as rs13425999, within the RAS guanyl releasing protein 3 (RASGRP3) gene locus.^294^ Moreover, a polymorphism in LPAR1 (rs10980684) has been associated with increased interferon levels and the presence of anti-Ro and anti-Sm antibodies.^297^

In addition to its role in pDCs, LPA signaling exerts broader immunopathological effects in SLE. Higher ATX and LPA levels have been associated with increased production of antiphospholipid antibodies and heightened platelet activation, contributing to thrombotic complications.^298^ Furthermore, dysregulated LPA signaling has been linked to the neuropathic pain symptoms observed in SLE patients.^299^ Although mechanistic studies in macrophages, monocyte-derived dendritic cells, and T cells remain limited, available evidence indicates that LPA promotes macrophage activation, TNF secretion, T-cell chemotaxis, and immune synapse remodeling—processes that likely exacerbate autoimmune inflammation.^300,301^

#### LPA and multiple sclerosis

Multiple sclerosis (MS) is a chronic autoimmune disorder characterized by neuroinflammation, demyelination, and neuronal degeneration within the central nervous system and is driven primarily by autoreactive T and B cells infiltrating the brain and spinal cord.^302^ Increasing evidence implicates LPA signaling—especially via the ATX‒LPAR1 axis—as a central contributor to MS pathogenesis.^303^ Exogenous application of the LPC precursor LPA and ATX to sciatic nerves, spinal nerves, and dorsal root fibers induces demyelination, supporting the pathogenic role of LPA signaling in MS.^303^ Clinical studies have confirmed elevated ATX levels in the serum and cerebrospinal fluid of MS patients compared with controls.^304^

At the receptor level, LPAR1 is upregulated in peripheral blood mononuclear cells and macrophages during MS relapse and EAE progression, where it facilitates M1 macrophage polarization, chemokine release, microglial and astrocyte activation, and oligodendrocyte suppression, which are hallmarks of demyelination and neuroinflammation.^305,306^ Targeted inhibition of LPAR1 via PIPE-791 preserves oligodendrocyte function, limits cytokine toxicity, reduces glial activation, and enhances remyelination in vivo.^306^ LPAR5 also contributes to MS pathology, with its expression induced during demyelination and linked to neuropathic pain via microglial activation in the corpus callosum.^307^

Conversely, some findings suggest that the loss of LPA signaling may exacerbate MS. Decreased LPA levels or LPAR2 deficiency worsens clinical symptoms in MS patients and EAE mice,^308^ whereas the administration of 2-carbacyclic phosphatidic acid (2 ccPA), an LPA analog, alleviates neuroinflammation and disease burden.^309^ These context-dependent outcomes underscore the dual nature of LPA signaling in MS, exerting either pathogenic or protective effects depending on the receptor subtype, immune environment, and disease stage.

#### LPA and systemic sclerosis

Systemic sclerosis (SS), or scleroderma, is a complex and heterogeneous autoimmune disorder characterized by vasculopathy, inflammation, and progressive fibrosis affecting the skin and internal organs, such as the kidneys, lungs, heart, esophagus, and bowel.^310^ Clinical symptoms include microvascular and macrovascular dysfunction, ischemia, vasoinstability, and the presence of specific autoantibodies, such as anti-Th/To, anti-topoisomerase I, anti-centromere, and anti-RNA polymerase III.^311^

LPA signaling has emerged as a key driver of SS pathogenesis. Cellular injury and chronic inflammation upregulate ATX, leading to increased LPA synthesis, which promotes fibrosis and inflammation.^312^ Elevated levels of circulating LPA, specifically LPA 20:4, are frequently observed in SS patients, with contributions from mast cells, platelets, and endothelial cells.^5^ A self-sustaining amplification loop in which LPA-induced IL-6 expression in dermal fibroblasts upregulates ATX expression, further amplifying fibrotic signaling, has been described.^7^ Fibroblasts from SS patients also show increased α-SMA expression and activity of the LPA-induced chloride channel ICI_LPA_, supporting a role in myofibroblast activation.^313^ LPA may also exacerbate SS-related vascular dysfunction through effects on endothelial remodeling.^314^

### LPA signaling in inflammatory diseases

**Fig. 5:**
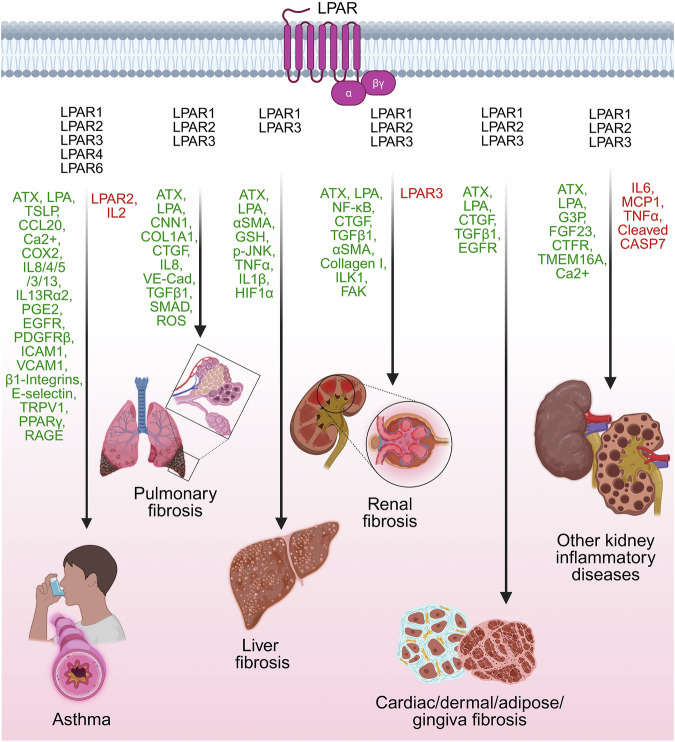
LPA signaling in the pathogenesis of inflammatory diseases. The figure elaborates on the role of LPA-LPAR signaling in the pathobiology of diverse inflammatory diseases, including asthma, pulmonary fibrosis, liver fibrosis, renal fibrosis, other fibrosis conditions and inflammatory diseases of the kidney. LPA receptors involved in each functional pathway are represented in black text, the upregulated signaling mediators are represented in green text, and the downregulated signaling mediators are represented in red text. Expanded abbreviations for signaling mediators are provided in the manuscript. The figure was created via BioRender.com under an academic license

LPA has been associated with numerous inflammatory diseases, such as asthma; fibrosis of the liver, lungs and kidney; and numerous kidney diseases (Fig. 5).

#### LPA and asthma

Asthma is a chronic inflammatory disease characterized by Th2-dominant immune responses. While the ATX–LPA axis is not essential for normal lung physiology, it plays a pathogenic role in asthma by mediating mast cell degranulation, eosinophil recruitment, epithelial barrier disruption, and airway hyperresponsiveness.^315,316^ Bronchoalveolar lavage (BAL) fluid from asthmatic patients and murine models has elevated levels of polyunsaturated LPA species and ATX,^317,318^ which preferentially activate proinflammatory pathways. Although the reason for the preferential upregulation of polyunsaturated LPA remains unclear, it may reflect the selective activation of downstream proinflammatory pathways. LPA signaling promotes asthma pathogenesis through multiple cellular targets, including airway epithelial cells (AECs), immune cells, smooth muscle cells (SMCs) and endothelial cells.

##### AECs

AECs, which interface with allergens and environmental stimuli, express LPARs and respond to LPA stimulation by secreting proinflammatory cytokines such as thymic stromal lymphopoietin (TSLP) and CCL20, promoting the recruitment of inflammatory immune cells.^319^ Upregulated LPAR1 and downregulated LPAR2 expression in AECs correlate with increased production and secretion of TSLP, IL-8, CCL20, and prostaglandin E2 (PGE-2) and increased expression of COX-2 and IL-13Rα2 through the PLC–PKCδ–p38MAPK–NF-κB signaling pathway.^319–322^ LPA also activates the PLD2–PKCζ–EGFR–ERK1/2–C/EBPβ pathways, stimulating the ERK1/2 and CCAAT/enhancer-binding protein beta (C/EBPβ)-mediated transcription of inflammatory mediators.^321,323^ The activation of PKCδ and PKCζ also promotes c-Met translocation to the cell membrane, disrupting airway epithelial barrier integrity via Rho-ROCK signaling.^324,325^

##### Immune cells

LPA enhances the chemotaxis of dendritic cells, monocytes, macrophages, T cells, and eosinophils.^315^ Depending on the activation state, LPA alters IL-2 expression and induces IL-13 production in Th2 cells, amplifying allergic inflammation.^326,327^ LPA also induces IL-13 production in Th2 cells and promotes mast cell maturation, further amplifying allergic inflammation.^327–329^

##### Smooth muscle cells

LPA-Rho-ROCK signaling increases calcium sensitization, enhancing muscarinic agonist–mediated airway smooth muscle contraction while impairing β2-adrenergic relaxation, leading to airway hyperresponsiveness.^329,330^ LPA also induces cytoskeletal remodeling and proliferation of bronchial smooth muscle cells, contributing to airway remodeling.^331–333^

##### Endothelial cells

Pulmonary arterial endothelial cells express LPAR2, LPAR6, LPAR1, LPAR3, and LPAR4 in descending order.^334^ The LPA-LPAR1 and LPA-Gαi–PI3K–Akt pathways disrupt endothelial barrier function via actin cytoskeleton remodeling, facilitating immune cell infiltration.^335–337^ LPA also upregulates adhesion molecules such as ICAM-1, VCAM-1, and E-selectin, promoting eosinophil and neutrophil infiltration into the lungs.^338^

LPA also engages noncanonical receptors expressed in airway and immune cells, such as TRPV1, PPARγ, and receptor for advanced glycation end products (RAGE), further modulating immune responses and bronchoconstriction.^339–341^ LPA-TRPV1 signaling in petrosal neurons activates the carotid body, promoting vagal-mediated bronchoconstriction.^341,342^ Although direct evidence for LPA-PPARγ and LPA-RAGE activation is lacking, the established roles of these receptors in dendritic cell migration and Th2 responses suggest the possible augmentation of these responses by LPA.^339–341^

While predominantly proinflammatory, LPA can also induce anti-Th2 mediators such as PGE2 in epithelial cells.^322,343,344^ Notably, LPAR2-deficient mice exhibit worsened inflammation in asthma models, suggesting receptor-specific protective roles.^345,346^ These discrepancies may arise from differences in LPA species composition, receptor subtype engagement, disease models, or genetic backgrounds. Together, these findings position LPA signaling as a critical orchestrator of airway inflammation, hyperresponsiveness, and remodeling in asthma.

#### LPA and fibrosis

Fibrosis, which is characterized by excessive ECM deposition, disrupts organ architecture and function, often following chronic inflammation. LPA has emerged as a key fibrotic driver across organs, including the lungs, liver, kidneys, skin, heart, and adipose tissue.^347^

##### LPA and pulmonary fibrosis

ATX and LPA levels are elevated in the fibrotic lungs of both humans and rodents.^348^ In idiopathic pulmonary fibrosis (IPF), a chronic interstitial lung disorder, ATX and LPA levels are increased in the BAL fluid of IPF patients.^36^

LPA production that drives IPF pathogenesis occurs not only through ATX-dependent mechanisms but also through ATX-independent pathways, such as those involving PLDs or PLAs.^6^ LPA signaling orchestrates IPF progression by regulating alveolar epithelial cells, endothelial cells, fibroblasts, and immune cells. LPAR1 expression is upregulated in lung fibroblasts, bronchial epithelial cells, and endothelial cells during IPF.^335^ LPA-LPAR1 signaling promotes fibroblast recruitment, vascular leakage, epithelial apoptosis, fibroblast survival, and profibrotic activation.^40,335,349,350^ LPA-LPAR1 signaling promotes bone marrow–derived mesenchymal stem cell differentiation into myofibroblasts, enhancing the secretion of ECM remodeling factors that promote pulmonary fibrosis.^351^ LPA also activates alveolar and interstitial macrophages to sustain chronic inflammation.^352–354^

In addition to LPAR1, LPAR2 contributes to pulmonary fibrosis via the induction of TGF-β1, which promotes fibroblast proliferation and the apoptosis of alveolar and bronchial epithelial cells.^355^ Downstream, LPAR2–Gαi/O signaling activates the PI3K–Akt and Ras–MAPK–ERK1/2–p38 pathways to upregulate TGF-β, which transactivates the SMAD cascade, inducing the expression of fibrotic genes such as CNN1 and COL1A1.^355^ In parallel, LPAR2–Gαq signaling drives epithelial apoptosis through mitochondrial ROS generation and caspase-3 activation while also enhancing fibrosis through the Rho–ROCK-αvβ6 integrin-TGF-β pathway.^356^

Consistent with these findings, genetic deletion of LPAR1/2 or ATX in bronchial epithelial cells or alveolar macrophages reduces vascular leakage, fibroblast recruitment, inflammatory cell infiltration, and ECM remodeling in pulmonary fibrosis mouse models.^6,335,352,355,357^ To further substantiate the role of LPAR1/2 in pulmonary fibrosis, pharmacological inhibition of LPAR1 (e.g., Ki16425, AM966, and BMS-986020) attenuated fibrosis and inflammatory cytokine production in mice, and BMS-986020 demonstrated clinical benefit in IPF patients in phase II trials.^335,350,358^ Similarly, LPAR1/3 inhibition with VPC12249 ameliorated radiation-induced lung fibrosis.^359^

##### LPA and liver fibrosis

Liver fibrosis is a progressive response to chronic injury and is characterized by the activation of hepatic stellate cells (HSCs), their differentiation into myofibroblasts, and the deposition of ECM components.^360^ LPA plays a central role in this cascade by promoting HSC proliferation, survival, and the transition of HSCs to ECM-secreting myofibroblasts.^361^

Clinical studies have shown that elevated serum ATX levels in patients with liver fibrosis and cholestasis-associated pruritus correlate with fibrosis severity and poor overall survival.^361,362^ An increase in ATX stems from both diminished hepatic clearance by sinusoidal endothelial cells and increased ATX production by activated hepatocytes.^363,364^ Consequently, ATX upregulation drives increased LPA production, amplifying profibrotic signaling without necessarily initiating fibrosis.^20,364^ Consistent with these findings, plasma LPA levels are markedly increased in both experimental models and patients with hepatitis C virus (HCV)-induced fibrosis.^365^ The increased ATX-LPA axis activity leads to the expression of prefibrotic proteins such as α-SMA, myofibroblast differentiation, cytoskeletal remodeling and liver fibrosis.^364^ Functionally, LPA activates the MAPK and Rho–ROCK pathways in HSCs, enhancing their proliferation, cytoskeletal remodeling, apoptosis resistance, and ECM deposition.^366^ It also triggers the pro-JNK and oxidative stress pathways, increasing TNF-α and IL-1β secretion, thereby linking inflammation to fibrogenesis.

In HCV-related disease, LPA-LPAR1/3 signaling stabilizes HIF-1α via PI3K activation, promoting viral replication and fibrotic progression toward hepatocellular carcinoma (HCC).^360^ Furthermore, LPA-LPAR1 signaling robustly upregulates TGF-β1, α-SMA, and CTGF, establishing a feedforward loop that sustains fibrosis.^367^

##### LPA and renal fibrosis

Renal fibrosis, a defining feature of progressive kidney disease, involves excessive ECM accumulation and tubular atrophy, leading to decreased renal function. Emerging evidence highlights a central role for LPA signaling, particularly via LPAR1 and LPAR2, in driving renal fibrosis.

In experimental models of unilateral ureteral obstruction (UUO), a well-established model of renal fibrosis, both renal LPA and LPAR1 levels are markedly upregulated, whereas LPAR3 expression is downregulated.^368^ LPA-LPAR1 signaling activates the Rho‒ROCK pathway in proximal tubular cells, promoting the expression of profibrotic cytokines such as CTGF and TGF-β, which stimulate fibroblast proliferation and myofibroblast differentiation via Ras–ERK1/2, promoting ECM deposition.^349,368^

LPA-LPAR1 signaling also facilitates macrophage infiltration into the kidney, further amplifying inflammatory and fibrotic pathways.^349,368^ Elevated LPAR1 expression has similarly been observed in renal interstitial fibrosis in nephrotoxic serum nephritis models with increased LPA levels following subtotal nephrectomy-induced renal fibrosis.^368–370^ LPA-LPAR2 signaling also contributes to renal fibrosis following ischemia‒reperfusion injury (IRI) through the activation of Gαq-Rho-ROCK pathways to induce TGF-β expression and fibroblast activation.^371^

In HIV-associated nephropathy, LPA–LPAR1/3 signaling modulates glomerular and tubular epithelial cells by activating NF-κB, increasing the expression of fibrotic markers such as α-SMA, CTGF, fibronectin, and collagen I, which is accompanied by the phosphorylation of key effectors such as PI3K, Akt, p38 MAPK, ERK, ILK-1, and FAK, driving renal nephropathy and fibrosis.^372^

##### LPA and other fibrosis conditions

Beyond the lungs, liver, and kidneys, LPA signaling contributes to fibrosis in tissues such as the skin, adipose, heart, and gingiva. In murine models of dermal fibrosis, genetic deletion of LPAR1, but not LPAR2, significantly reduces fibrosis by downregulating CTGF and TGF-β1, implicating LPA–LPAR1 in cutaneous fibrotic remodeling.^7,373^ Similarly, LPA–LPAR1 promotes adipose tissue fibrosis, enhancing ECM deposition and structural remodeling under metabolic stress.^268^ LPA signaling is also implicated in gingival fibromatosis, a condition characterized by excessive connective tissue buildup. In gingival epithelial cells, LPA enhances CTGF/CCN2 expression, identifying LPA as a potential driver of oral fibrotic changes.^374^

In contrast, LPAR3 appears to mediate antifibrotic effects in the heart. In models of cardiac fibrosis, osteoglycin, rather than LPA, activates LPAR3–Gα12/13–Rho–ROCK signaling, suppressing EGFR transactivation and myocardial fibroblast proliferation, suggesting context-specific protective roles for certain LPA receptors.^375^

#### LPA and kidney diseases

LPA signaling plays a multifaceted role in kidney pathologies, including acute kidney injury (AKI), chronic kidney disease (CKD), DN, and renal fibrosis. While DN and fibrosis have been addressed earlier, this section focuses on broader contributions of LPA to renal pathophysiology. In AKI, metabolic stress elevates glycerol-3-phosphate (G3P) in the renal cortex, which is transported to the bone marrow and converted to LPA via GPAT2. The resulting LPA activates LPAR1, increasing the level of fibroblast growth factor 23 (FGF23), a marker of AKI severity linked to phosphate imbalance, immune dysregulation, cardiovascular risk, and poor prognosis.^376,377^ Although some evidence points to a Gq/11α-like extralarge Gα protein (GXLα)-IP3-PKC pathway in FGF23 induction,^378^ its role in LPA signaling remains unclear. In CKD, the plasma levels of LPA, specifically LPA (16:0) and LPA (18:1), are elevated and partially reduced by hemodialysis.^379^ Interestingly, some studies report preferential elevation of urinary LPA, suggesting tissue-specific dynamics in LPA production and clearance during CKD progression.^370,380^

In line with these findings, LPAR1 expression is upregulated in murine models of CKD,^368,381^ where it drives profibrotic and proinflammatory pathways. In the UUO model, LPA-LPAR1 signaling enhances macrophage infiltration into the kidney, exacerbating inflammation and fibrosis.^370,380^ Pharmacologic inhibition of LPAR1/3 with BMS002 significantly improved renal function by reducing podocyte loss, preserving tubular epithelial integrity, and improving glomerular filtration rates.^382^ However, in contrast, subtotal nephrectomy models demonstrated reductions in ATX, LPAR1-3, and LPP expression, reflecting potential stage- or model-specific differences in LPA pathway activity.^370^ LPA also regulates mesangial cell proliferation, expanding its role in glomerular disorders through a pathway involving LPAR1 activation of Rac and MAPK.^383^

Notably, LPA elicits context-dependent dual effects in the kidney. In IRI models, LPA (18:1), via LPAR2, protects renal function by reducing apoptosis, complement activation (C3/C6), and inflammatory cytokines (IL-6, MCP-1, TNF-α), whereas LPAR1/3 signaling exacerbates renal damage.^384,385^

### LPA signaling in cancer pathobiology

**Fig. 6:**
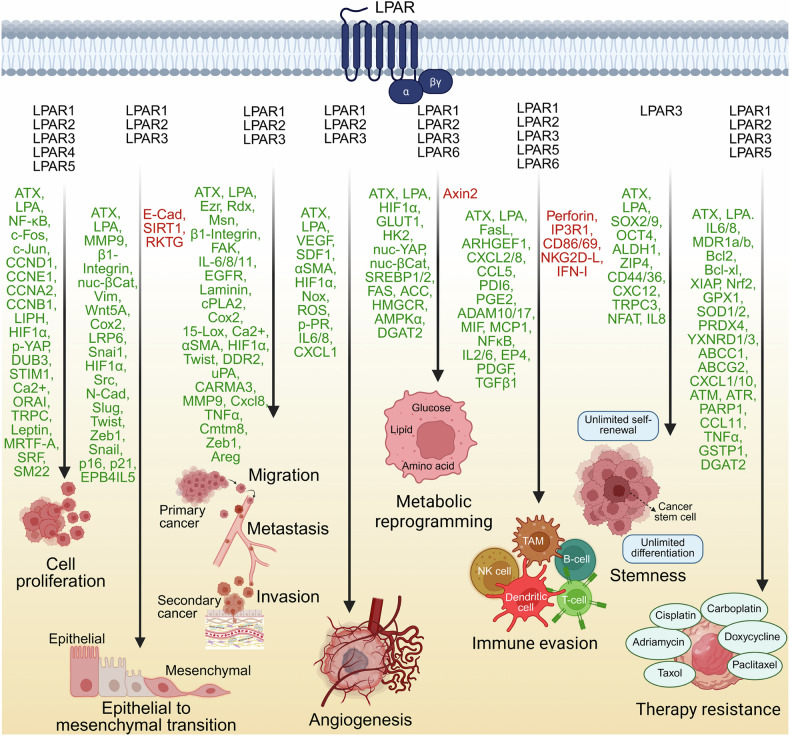
LPA signaling in the pathobiology of cancer. The figure summarizes the role of LPA-LPAR signaling in the pathobiology of cancer by tracing its contribution to cancer hallmarks such as cell survival and proliferation, epithelial to mesenchymal transition, migration, metastasis, invasion, angiogenesis, metabolic reprogramming, immune evasion, therapy resistance and cancer stemness. LPAR sub-types involved in each functional pathway are represented in black, the upregulated signaling mediators are represented in green, and the downregulated signaling mediators are represented in red. [LPAR LPA receptor, B-cell B lymphocytes, T-cell T lymphocytes, NK cells natural killer cells, TAM tumor-associated macrophages. The abbreviations representing the signaling mediators are detailed in the main text]. The figure was created via BioRender.com under an academic license

LPA plays a critical role in cancer pathogenesis by stimulating diverse oncogenic processes, including cell proliferation, survival, differentiation, stress-induced antiapoptotic signaling, EMT, migration, invasion, metastasis, immune evasion, metabolic reprogramming, the DNA damage response, neovascularization, angiogenesis, and resistance to chemotherapy and radiotherapy (Fig. 6). These functions collectively drive cancer initiation, progression, and therapeutic resistance.

LPA is actively synthesized by cancer cells as well as by several cell types in the TME. Among the different cellular components of the TME—fibroblasts, endothelial cells, adipocytes, stellate cells, T cells, B cells, macrophages, NK cells, neutrophils and dendritic cells—stromal cancer-associated fibroblasts, endothelial cells, adipocytes, and macrophages synthesize and secrete LPA into the TME.^386–388^

#### Cell Survival and Proliferation

A key oncogenic function of LPA is the promotion of cancer cell survival and proliferation, which is achieved through the regulation of cell cycle genes, the activation of prosurvival signaling, metabolic remodeling, and apoptotic resistance.

LPA stimulates cancer cell proliferation primarily by regulating key cell cycle genes in many different cancers, although receptor engagement varies. In colon cancer, LPAR1/2/3 activation triggers Rho–ROCK and STAT3 signaling, increasing the expression of Cyclin E1, Cyclin A2, and Cyclin B1 and promoting S and G2/M phase progression.^1^ In ovarian cancer (OC), LPA signals through LPAR2 to stimulate NF-κB, c-Fos, and c-Jun, driving Cyclin D1 expression and G1/S phase progression.^389^ PI3K signaling has emerged as a central conduit for LPA-driven proliferation across multiple cancers. In pancreatic ductal adenocarcinoma (PDAC), K-Ras–induced lipase H (LIPH) expression increases LPA production, activating LPAR1/3–PI3K–Akt, stabilizing HIF-1α, and inhibiting YAP phosphorylation to drive growth.^390^ In thyroid carcinoma, LPA-LPAR5 signaling stimulates the PI3K p110β subunit, leading to Akt and p70S6K1 activation and the engagement of the mTOR pathway for cell proliferation and survival.^391^ Similarly, LPA-LPAR1 activation supports esophageal squamous cell carcinoma (ESCC) proliferation via PI3K-Akt signaling.^392^ In gastric cancer, LPA-LPAR3 transactivates EGFR–PI3K–mTOR signaling, upregulating the deubiquitinase DUB3, stabilizing geminin, and promoting S-phase progression by preventing DNA damage-induced apoptosis.^393^

In addition to its role in classical mitogenic pathways, LPA modulates intracellular calcium dynamics to stimulate proliferation. LPA-Gαq/11-PLC-STIM1 (stromal interaction molecule 1) signaling enhances Ca²⁺ entry via ORAI and TRPC (transient receptor potential cation channel) channels, leading to hyperpolarization and activation of proliferative signals, particularly in OC cells.^44^ Inflammatory cues further intertwine with LPA-driven proliferation. In HCC, LPA-LPAR2 signaling induces p38MAPK activation, enhancing CEBP- and HIF-1α-mediated transcription of leptin, a proinflammatory mediator that fuels tumor growth in vivo.^394^ In HCC, LPA-LPAR4 signaling stimulates filamin A phosphorylation and myocardin-related transcription factor A (MRTF-A) transcriptional activation, promoting the expression of serum response factor (SRF) and SM22/transgelin to stimulate proliferation in HCC.^395^ These findings highlight LPA as a master regulator of cancer cell proliferation that acts through coordinated control of cell cycle regulators, survival kinases, inflammatory mediators, and calcium signaling pathways to sustain tumor growth.

Taken together, these findings reveal a common signaling architecture underlying LPA-mediated tumor cell growth. Despite being activated through distinct LPAR subtypes, LPA signaling consistently converges on core effectors such as PI3K–Akt, mTOR, and YAP/HIF-1α, which integrate cell cycle regulation, survival signaling, and metabolic adaptation, sustaining uncontrolled tumor growth across cancers.

#### Epithelial-to-mesenchymal transition (EMT)

EMT is a pivotal biological process through which epithelial cells acquire mesenchymal properties, characterized by the loss of cell‒cell adhesion marked by the downregulation of epithelial markers (E-cadherin, cytokeratins, desmoplakin, and occludin) and the gain of mesenchymal traits involving N-cadherin, vimentin, fibronectin, Snail, Slug, and Twist. This transition endows cancer cells with enhanced migratory and invasive capabilities, contributing to metastasis, therapy resistance, and disease progression.^396^

LPA is a potent inducer of EMT across cancer types. In OC, LPA upregulates MMP-9, promoting E-cadherin shedding and junctional destabilization. This triggers β-catenin nuclear translocation, activating Wnt/β-catenin signaling and EMT transcription. Simultaneously, LPA enhances the expression of vimentin, WNT5A, PTGS2 (Cox-2), LRP6, and SNAI1, reprogramming F-actin and integrins to reinforce mesenchymal transition.^12^

LPA similarly orchestrates EMT programs in other malignancies. In breast cancer, LPA-RAGE-PKB axis activation induces key EMT transcription factors, including Slug, Snail, and Twist, enhancing tumorigenicity.^397^ In HCC, the LPAR1–PI3K–Akt–mTOR–Skp2–p27Kip1 axis modulates EMT markers such as E-cadherin, N-cadherin, vimentin, fibronectin, Snai1, and Twist, which drive HCC progression.^50^ In addition, the loss of RKTG, a tumor suppressor, in HCC sensitizes HCC cells to LPA-induced Akt and GSK3β activation, stabilizing p53 and promoting p16 and p21 expression, further linking LPA to EMT induction.^52^ In gastric cancer, LPA-LPAR2 signaling activates the Notch pathway to drive EMT and invasiveness.^398^ In clear cell renal carcinoma, LPA-LPAR2 utilizes the Gα12-Exchange factor for Arf6 (EFA6)-Adenosine ribosyl factor 6 (Arf6)-Arf-GTPase activating protein 1 (AMAP1)-Erythrocyte Membrane Protein Band 4.1 Like 5 (EPB41L5) to orchestrate cytoskeletal remodeling and integrin dynamics to drive EMT.^399^

While LPA engages diverse upstream inputs to induce EMT, these pathways converge on a shared transcriptional output. LPA-driven EMT utilizes various upstream cues, including the Wnt/β-catenin, PI3K-Akt, Notch, and HIF-1α-Src axes, but converges on transcriptional regulators such as Snail, Slug, Twist, and ZEB1, which orchestrate epithelial suppression and mesenchymal transition, enabling cellular plasticity and metastatic potential.

#### Migration

LPA-induced cancer cell migration is driven by cytoskeletal reorganization, chemotaxis, and integrin signaling. In OC, LPA activates Gα12/13-mediated RhoA signaling through LPAR1 and LPAR2, promoting the phosphorylation and translocation of Ezrin Radixin Moesin (ERM) proteins to cortical protrusions, as indicated by colocalization studies with F-actin, which enhances motility.^400^ LPA also upregulates β1-integrin, increasing adhesion and migration, a process reinforced by Gαi–H-Ras–MEKK1 signaling and FAK redistribution.^401^ In addition, LPA promotes IL-8 secretion via Gαi-mediated EGFR transactivation or Gα12/13-mediated EGFR-independent AP1 activation and/or Gαq-PLC-PKC-driven NF-κB signaling, collectively enhancing OC cell migration.^402^ In PDAC, LPA activates PI3K–Akt and ERK1/2–p38 MAPK to drive migration and dissemination, whereas Gαi/o–Ras–Rac1–RhoA–ERK signaling further reinforces PDAC cell motility.^403^ In HCC, LPA-LPAR3-Gαi/o-Akt-ERK1/2 signaling regulates tumor cell motility. In glioblastoma, microglia-derived LPA affects LPAR1 on tumor cells to promote their migration and progression.^404^

In addition to its direct effect on tumor cells, LPA modulates the TME to promote cancer cell migration. In OC, laminin–induced LPA production and an LPAR3–PI3K autocrine loop enhance migration despite the spatial relationships underlying LPA and laminins within the OC TME.^405^ LPA further augments migration toward laminin via LPAR1/3–MEK–ERK–cPLA2, increasing COX and 15-LOX expression in a migration assay.^406^ The use of pharmacological inhibitors of LPAR1/3 (Ki16425), cPLA2/iPLA2 (AACOCF3), iPLA2 (HELSS), LOX (nordihydroguaiaretic acid), COX-2 (NS398), COX-1 (SC-560), and 15-LOX (PD146176) validated this signaling axis.^407^ Although the mechanism by which COX and 15-LOX promote OC cell migration to laminin is not clear, it is possible that the COX-2/LOX-mediated release of eicosanoids promotes migration. In OC, increased LPA promotes the migration of human adipose-derived mesenchymal stem cells (HAMSCs) in the TME via the LPAR1–MEK–ERK and RhoA–ROCK signaling pathways.^408^ Since LPA can be synthesized and released by multiple cell types in the TME, these findings further underscore the role of the TME in promoting HAMSC migration and cancer progression. In HCC, tumor-secreted LPA induces the transdifferentiation of fibroblasts, facilitating tumor cell invasion.^409^ LPA also enhances migration across pancreatic, colorectal, colon, and oral cancers via EGFR transactivation-Akt-ERK signaling.^50^

Despite tissue- and receptor-specific variations, LPA-directed migration is built on a core set of motility-enabling signals. Across tumor types and receptor subtypes, LPA signaling converges on RhoA–ROCK, PI3K–Akt–ERK, and NF-κB, coordinating cytoskeletal rearrangement, focal adhesion turnover, and promigratory gene expression that collectively drive directional cell motility.

#### Invasion

LPA-induced invasion involves both directed cell movement and degradation of the ECM. Under hypoxic conditions, LPA increases cytoplasmic PLA2 activity via the HIF-1α pathway, promoting invasion toward collagen I and enhancing metastasis in vivo.^410^ LPA also enhances HIF-1α through PI3K–Akt–mTOR, driving Twist and Discoidin Domain Receptor 2 (DDR2) expression to promote invasive phenotypes.^411^ LPA-induced COX-2 expression via Gαi-cSrc-EGFR transactivation and Ras/MAPK–ERK/MEK signaling promotes eicosanoid-mediated OC cell invasion.^67^ LPA also induces uPA, a serine protease that aids in ECM degradation and invasion, via multiple pathways, including the Gαi-Ras-Raf-NF-κB,^412^ PKCα-CARMA3 (CARD-recruited membrane-associated protein 3) axes,^413^ and p38MAPK signaling pathways.^414^

In PDAC, LPAR3 expression is upregulated, and the LPA-LPAR3-mediated signaling pathway promotes invasion.^415^ In HCC, the LPA-LPAR1 axis drives invasion through the PI3K/Akt and PKCδ-p38MAPK pathways, which upregulate MMP-9 expression.^416^ In prostate cancer, LPA activates Akt and IκBα, inducing RhoA and NF-κB, which stimulate invadopodia formation and invasion.^50^ In glioblastoma, LPA-LPAR-Gαio signaling activates both Rho-ROCK and Ca2 + -ERK1/2 to promote cancer cell invasion.^417^

LPA-induced invasion is governed by a convergence of signaling modules that promote matrix degradation and tumor infiltration. Diverse LPA-initiated signaling routes funnel into a common output through effectors such as RhoA, HIF-1α, NF-κB, and MAPKs, which regulate invadopodia formation, MMP/uPA expression, and hypoxia-induced adaptation, facilitating matrix degradation and invasive spread.

#### Metastasis

LPA promotes metastasis across multiple cancers by integrating proinvasive, prosurvival, and TME-remodeling mechanisms. In OC, peritoneal metastasis, elevated LPA in peritoneal fluid supports cell shedding, anoikis resistance, and collagen I–mediated adhesion, driving peritoneal invasion via MEK/ERK and Akt signaling.^418^ In renal carcinoma, the LPAR2–MAPK–NF-κB axis induces CXCL8, IL-6, and TNF, enhancing migration and lung metastasis.^419^ In PDAC, LPA-LPAR1 signaling stabilizes CKLF, similar to the MARVEL transmembrane domain containing 8 protein (CMTM8), activating β-catenin to drive lung metastasis. The observation that ATX deletion reduces metastasis underscores the critical role of LPA in pancreatic cancer metastasis.^420^

In osteosarcoma, cancer-induced platelet activation elevates LPA levels, which in turn signal via LPAR1 to promote pulmonary metastasis.^421^ In breast cancer models, LPAR3–Gαi/o–Rho–YAP signaling induces Zeb1 and AREG, facilitating fibroblast-to-CAF conversion and TME remodeling and promoting lung metastasis.^422^ Breast tumors with LPAR3 overexpression exhibit enhanced lymph node and lung metastases. Consistent with these findings, breast cancers with LPAR3 overexpression exhibit heightened metastatic spread to regional lymph nodes and lungs.^423^ Finally, in prostate cancer, LPA induces IL-6 expression, which promotes osteoclast differentiation and bone resorption, favoring bone metastasis.^50^

Although receptor usage and downstream effectors vary by cancer type, LPA-driven metastasis converges on a set of common outputs. These include cytokine-mediated TME modulation, anoikis resistance, and activation of the β-catenin, NF-κB, and YAP pathways, collectively orchestrating tissue-specific metastatic tropism and systemic dissemination.

#### Tumor angiogenesis

Tumor angiogenesis, the formation of new blood vessels from preexisting vasculature, is essential for tumor growth, invasion, and metastasis. Among the proangiogenic mediators, vascular endothelial growth factor (VEGF) plays a central role, and LPA signaling has emerged as a potent regulator of VEGF-driven neovascularization across multiple cancer types.

In OC, LPAR1 activation in human adipose-derived mesenchymal stem cells (HAMSCs) promotes the secretion of VEGF, SDF-1, and α-SMA via the ROCK‒PLC‒PI3K‒ERK pathway.^408^ LPA also stabilizes HIF-1α through the PI3K–Akt–mTOR–p70S6K pathway and MAPK, increasing VEGF promoter activation. In prostate cancer models, elevated LPA levels activate LPAR1/3 signaling, inducing eukaryotic initiation factor 2 alpha (eIF-2α) phosphorylation and calreticulin-mediated VEGF-C mRNA stabilization, thereby promoting lymphangiogenesis.^424^ Parallel pathways involving LPAR1/3-PLC–PKC–Nox–ROS signaling also upregulate VEGF-C and angiogenic responses in prostate cancer cells.^425^ In colon cancer, LPAR1/2 activation enhances VEGF production and angiogenesis,^426^ whereas in glioblastoma, LPAR1–PKCα signaling phosphorylates the progesterone receptor, increasing VEGF transcription and angiogenesis.^306^ The causal link between LPA signaling and VEGF expression is further supported by studies showing that pharmacological inhibition of LPA synthesis attenuates tumor angiogenesis in papillary thyroid carcinoma models.^427^

In addition to VEGF-mediated mechanisms, LPA modulates proangiogenic cytokines and chemokines. In OC, LPA-LPAR2 signaling via JNK and p38MAPK along with Gαi-PI3K-Akt signaling induces IL-6 and IL-8 expression and secretion, driving angiogenesis.^428^ LPA-LPAR2 signaling also induces the expression of GROα/CXCL1, a chemokine that binds to CXCR2 receptors on endothelial cells to initiate angiogenic signaling. Elevated GROα/CXCL1 levels in OC ascites and serum samples from OC patients strongly correlate with high LPA levels, reinforcing its role in TME neovascular signaling.^429^ The release of angiogenic cytokines and chemokines such as IL-6, IL-8 and CXCL1 as well as VEGF by OC cells promotes the activation of endothelial cells that drive neovascularization.

Collectively, these findings reveal a unifying mechanism by which LPA signaling promotes tumor angiogenesis across cancers. Through both VEGF-driven transcriptional activation and cytokine/chemokine secretion, LPA-LPAR signaling converges on endothelial activation, thereby orchestrating a robust neovascular program essential for tumor growth and metastatic potential.

#### Metabolic reprogramming

Metabolic reprogramming enables cancer cells to sustain proliferation and resist stress, with LPA signaling emerging as a key regulator of glycolytic and lipogenic shifts in the TME.

##### Glycolytic reprogramming

LPA signaling promotes the Warburg effect, a shift toward aerobic glycolysis even in the presence of oxygen, across multiple cancer contexts. In OC, LPA induces pseudohypoxia through Gαi2-Rac-mediated upregulation of HIF-1α, which in turn drives the expression of the glucose transporter GLUT1 and the glycolytic enzyme hexokinase II (HKII), facilitating a glycolytic shift.^430^ In addition, OC cell-derived LPA in the TME transforms normal fibroblasts into CAFs and induces a HIF-1α-dependent glycolytic shift in these CAFs, further supporting tumor metabolism.^431^ In PDAC, the LIPH-mediated increase in LPA levels activates the LPAR1/3-PI3K-Akt-HIF-1α axis. This signaling axis, along with YAP1 nuclear translocation, promotes the transcriptional upregulation of key glycolytic enzymes such as HK2 and GLUT1.^390^ In gastric cancer, LPA-LPAR2 signaling triggers β-catenin nuclear localization via GSK-3β phosphorylation and Axin2 degradation, enhancing both glycolysis and oxidative phosphorylation (OXPHOS).^432^ In HCC, LPA-LPAR6 signaling promotes glycolysis and suppresses OXPHOS, supporting cancer growth and chemoresistance.^433^

##### Lipogenic reprogramming

LPA signaling also fuels lipid biosynthesis, a critical adaptation for rapidly proliferating cancer cells. In OC cells, LPA-LPAR2-Gα12/13-Rho signaling activates SREBP-1/2, inducing the expression of fatty acid synthase (FAS), acetyl-CoA carboxylase (ACC), and HMG-CoA reductase (HMGCR), key enzymes in lipid biosynthesis.^77^ In parallel, LPA-LPAR2-Gαq-PLC signaling dephosphorylates and inactivates AMP-activated protein kinase alpha (AMPKα), reducing the inhibitory phosphorylation of ACC and further enhancing lipogenesis. Together, these pathways fuel tumor growth by increasing lipid availability for membrane synthesis and energy storage.^77^

By integrating HIF-1α, β-catenin, and SREBP-dependent transcriptional programs, LPA signaling converges on dual reprogramming of glycolysis and lipogenesis, enabling tumors to adapt, grow, and resist therapy under metabolic constraints.

#### Immune evasion

LPA signaling subverts immune surveillance by modulating both adaptive and innate immune arms, creating an immunosuppressive TME that fosters progression and metastasis. Most of these effects are mediated by the LPA synthesized and secreted by primary immune cells within the TME, including tumor-associated macrophages (TAMs), T lymphocytes, and platelets.^59,434,435^

##### Adaptive immune suppression

LPA profoundly impairs T-cell–mediated immunity, blunting the cytotoxic activity essential for tumor clearance. In OC, LPA-LPAR5 signaling suppresses CD8⁺ T-cell function by reducing perforin release, thereby weakening cytotoxic immune responses.^436^ LPA also induces FasL-mediated apoptosis of activated T cells via the MEK-ERK1/2 pathway, conferring immune privilege to tumor cells.^35^ In melanoma and NSCLC, LPA–LPAR5 signaling inhibits T-cell infiltration, promotes PD-1 therapy resistance, and enhances inflammatory gene expression.^437–441^ LPA-LPAR5 signaling also promotes T-cell exhaustion, redirecting metabolism toward fatty acid oxidation and increasing oxidative stress, further dampening antitumor responses.^442^

LPA also disrupts adaptive humoral immune responses by impairing B-cell receptor (BCR) signaling in B lymphocytes. LPA, through the LPAR5-Gα12/13-ARHGEF1 pathway, interferes with IP3 receptor activity and intracellular Ca2+ release, preventing antigen-specific induction of CD86/69 expression on B cells and thereby impairing humoral immunity.^443^

##### Innate immune suppression

LPA also remodels innate immunity to support tumor growth. Innate immune responses are mediated mainly through macrophages, neutrophils, eosinophils, basophils, NK cells, mast cells, dendritic cells and platelets.

LPA in the TME and ascites of peritoneal cancer patients induces the differentiation and activation of macrophages, particularly those that transform monocytes into TAMs of the M2 phenotype, through the PI3K/Akt and JAK/STAT signaling pathways. TAMs, in turn, secrete additional LPA, creating a feedforward loop that sustains immune suppression and tumor growth.^444,445^ LPA also promotes the differentiation of CD11+ monocytes into F4/80+ macrophages via the Akt/mTOR pathway, a process regulated by PPARγ. These TAMs play key roles in ECM remodeling, which facilitates tumor invasion and immune evasion.^354^ TAM-secreted chemokines (CXCL8, CCL5, and CXCL2) recruit myeloid cells, whereas proteases such as PDI6 and ADAM10/17 cleave NKG2D ligands, undermining cytotoxic recognition by NK and CD8⁺ T cells.^446–449^ In mouse models of colon carcinoma, LPAR2 knockout reduces macrophage infiltration and delays tumor progression.^450^ LPA also suppresses NK cell cytotoxicity via LPAR2-Gαs-PKA-cAMP, reducing perforin release and impairing the killing of Burkitt’s lymphoma and melanoma cells.^451^ LPA also inhibits type I interferon (IFN I) production and immune responses by dendritic cells, mainly through the LPA-LPAR-PGE2-E-type prostanoid 4 receptor (EP4) axis, which compromises IFN-induced TLR-mediated antitumor immunity.^450^

In addition to macrophages, platelets are significant contributors of LPA in the TME118. LPA-activated platelets release protumorigenic mediators such as IL-6, PDGF, and TGF-β, promoting immune evasion.^452,453^ Activated platelets also shield circulating tumor cells (CTCs) from NK cell attack via TGF-β– and PDGF-mediated suppression of cytotoxicity.^452,454,455^

Interestingly, in contrast to its common immunosuppressive role, LPA has been shown to promote antitumor innate immune responses in colorectal cancer. In this context, the silencing of AGPAT4, which normally converts LPA into phosphatidic acid, leads to elevated LPA levels that stimulate M1 macrophage polarization via LPAR1/3–p38 MAPK–NF-κB signaling, increasing the release of proinflammatory cytokines (IL-1β, IL-6, and TNF-α) and facilitating CD4⁺/CD8⁺ T-cell infiltration and IFN-γ production, ultimately suppressing tumor growth.^51^ This highlights the receptor- and context-specific immunomodulatory functions of LPA signaling within the TME.

#### Cancer stemness

Cancer stem cells (CSCs) are a tumor subpopulation with self-renewal, pluripotency, and therapeutic resistance capabilities that drive tumor initiation, progression, metastasis, and recurrence.^456^ Emerging evidence implicates LPA signaling as a pivotal regulator of CSC maintenance across diverse cancers.

In OC, LPA promotes CSC phenotypes by upregulating core transcription factors such as OCT4, SOX2, and ALDH1, sustaining self-renewal and inhibiting differentiation.^59^ Activation of this pathway fosters self-renewal and suppresses differentiation, promoting aggressive tumor growth. Through PPARγ activation, LPA enhances the expression of ZIP4, a zinc transporter that is correlated with increased OCT4, ALDH1, and side population (SP) cells—hallmarks of CSCs.^457^ LPA also induces SOX9 via PPARγ, which in turn upregulates CD44 and promotes spheroid formation, further enriching the CSC pool.^59^ In addition, tumor-associated mesenchymal stem cells (MSCs) in the OC microenvironment secrete CXCL12 in response to LPA, activating CXCR4 on tumor cells and promoting hypoxia resistance and stemness.^457^

In triple-negative breast cancer (TNBC), LPA-LPAR3 signaling activates TRPC3 channels, leading to increased intracellular calcium levels, activation of the transcription factor NFAT, and upregulation of IL-8, which collectively promote the expansion of ALDH⁺ CSC populations.^458^ In estrogen receptor–positive (ER⁺) breast cancers, CSCs localize within arteriolar niches, where endothelial cell–derived LPA activates the LPA–LPAR–PKD-1–CD36 axis, supporting CSC growth and survival.^459^ Pharmacologic inhibition of ATX impairs CSC growth and maintenance in breast cancer models, substantiating the critical role of LPA in promoting cancer stemness and highlighting its therapeutic potential.^460^

Thus, LPA–LPAR signaling sustains cancer stemness by regulating transcriptional, metabolic, and paracrine circuits that reinforce self-renewal, hypoxia resistance, and niche-dependent growth

#### Therapy resistance

Therapy resistance remains a major challenge in cancer treatment, often leading to recurrence and poor prognosis. Increasing evidence implicates the ATX–LPA–LPAR axis as a central driver of chemo- and radioresistance across multiple tumor types, modulating survival signaling, drug efflux, metabolic rewiring, DNA repair, and immune evasion.^461^

##### Chemoresistance

LPA-mediated signaling confers chemoresistance through multiple, often overlapping pathways. In OC, LPA induces the expression of ZIP4 and promotes EGFR upregulation and transactivation, enhancing resistance to cisplatin and doxycycline therapies.^59,462^ LPA-LPAR2 signaling, via TRIP6 and Na + /H+ exchanger regulatory factor 2 (NHERF2), activates antiapoptotic signals that confer resistance to cisplatin and adriamycin,^463^ whereas PI3K–MEK–p38 signaling counters paclitaxel-induced apoptosis by suppressing caspase-3.^464^ LPA also promotes IL-8 secretion and upregulates MDR1, Bcl2 (B-cell lymphoma 2), Bcl-xl (B-cell lymphoma extralarge), and XIAP (X-linked inhibitor of apoptosis protein), enhancing chemoresistance via the PI3K–Akt and Ras–MEK–ERK pathways.^465^

In breast cancer, LPA-LPAR1 signaling activates the transcription factor Nuclear Factor Erythroid 2-Related Factor 2 (Nrf2), which drives the expression of antioxidant defense genes e.g., Glutathione Peroxidase 1 (GPX-1), Glutamate-Cysteine Ligase Modifier Subunit (GCLM), Superoxide Dismutase ½ (SOD1/2), Peroxiredoxin 4 (PRDX4), Thioredoxin Reductase 1 (TXNRD1/3), and multidrug transporters, such as ATP-Binding Cassette Subfamily C Member 1 (ABCC1) and ATP-Binding Cassette Subfamily G Member 2 (ABCG2), thus facilitating drug detoxification and efflux.^466^ In addition, LPA also reverses Taxol-induced G2/M cell cycle arrest and inhibits ceramide production via the PI3K pathway, preventing apoptosis.^467^ In PDAC, CAF-derived LPA activates NF-κB–CXCL1, driving immune evasion and resistance to TGF-β-targeted therapy, whereas LPA-LPAR2/3 signaling confers resistance to cisplatin.^468^

Additionally, in HCC, LPA-LPAR6 signaling reprograms glucose metabolism to augment glycolysis, driving resistance to the kinase inhibitor sorafenib.^433^ In renal cell carcinoma, LPA restores lipid metabolism via the MAPK-S6-DGAT2 pathway, undermining the efficacy of the mTOR inhibitor temsirolimus.^469^ In cervical cancer, LPA confers resistance to doxycycline by attenuating doxycycline-induced caspase-3-mediated apoptosis.^470^

##### LPA and radioresistance in cancers

LPA also orchestrates resistance to radiotherapy. In breast cancer patients and experimental models, LPA upregulates inflammatory mediators, including IL-6, TNF-α, CCL11, and CXCL10, enabling tumor cells to survive radiation-induced stress.^471,472^ LPA-LPAR1 signaling induces Nrf2, promoting the expression of DNA repair genes such as ataxia telangiectasia mutated (ATM), ATM and Rad3-related (ATR), and poly (ADP-ribose) polymerase 1 (PARP-1), thereby facilitating efficient repair of radiation-induced DNA damage.^466,473–476^ The LPA-LPAR2 axis has also been implicated in reinforcing DNA repair mechanisms critical for radioresistance.^474^ In glioblastoma, targeting LPA signaling with ATX inhibitors such as PF-8380 and BrP-LPA has shown promising results in overcoming resistance to radiotherapy and promoting radiosensitivity.^477,478^ Mechanistically, LPAR2 binds to Siva1, a proapoptotic protein activated by DNA damage, and prevents Siva1-Bcl-xL-mediated mitochondrial apoptosis by targeting Siva1 for degradation. Concurrently, the recruitment of TRIP6 and NHERF2 by LPAR2 activates prosurvival Akt and ERK1/2 signaling, enabling OC cells to withstand DNA damage induced by radiation and chemotherapy.^463,479^ Interestingly, pharmacologic activation of LPAR2 with LPAR2 agonists such as DBIB has been used to protect normal hematopoietic and GI cells during therapeutic irradiation, highlighting the context-specific dual role of LPA.^480,481^ Together, these findings underscore the central role of the LPA-LPAR axis in orchestrating chemoresistance and radioresistance through multifaceted mechanisms involving prosurvival signaling, metabolic adaptation, and DNA repair, identifying an actionable target to increase therapeutic efficacy.

## Therapeutic targeting of LPA signaling

Owing to its central role in cardiovascular, neurological, inflammatory, autoimmune, and metabolic disorders and in cancer pathobiology, the LPA signaling axis has emerged as a compelling therapeutic target.^1,50^ Elevated levels of ATX, LPA, LPARs, and LPPs in patient samples underscore its diagnostic and prognostic relevance across diverse disease settings. Therapeutic interventions have focused on three major strategies: ATX inhibition to reduce LPA production, LPAR antagonism to block receptor-mediated signaling, and the targeting of downstream effectors to disrupt disease-driving pathways. These approaches aim not only to halt disease progression but also to counteract therapy resistance and potentiate current treatments, ultimately improving clinical outcomes.

### ATX inhibitors

Multiple in vitro and in vivo studies, including genetic knockout models, have demonstrated that ATX inhibition can mitigate disease progression across a wide spectrum of pathological conditions. On the basis of their structural binding sites, ATX inhibitors are classified into five categories^482–484^: Class I, Class II, Class III, Class IV, and Class V, which bind to the orthosteric site, the hydrophobic pocket, the allosteric tunnel, the tunnel‒pocket hybrid, and the tunnel‒active site hybrid, respectively. Representative compounds include HA-130, HA-155, PF-8380, and 2 ccPA (Class I); PAT-494 and IOA-289 (Class II); UDCA (Class III); GLPG1690 and BBT-877 (Class IV); and CG-23 (Class V). These inhibitors have shown efficacy in preclinical models of cancer, inflammatory and autoimmune diseases, metabolic syndromes such as obesity-related cardiomyopathy, and neurological disorders.

For example, HA-155 has been shown to have inhibitory effects on cardiovascular diseases and obesity,^262,485^ whereas PF-8380 has been shown to have anticancer effects on PDAC,^486^ radiosensitization effects on glioblastoma,^478^ anti-inflammatory,^487^ and antiobesity effects.^177^ The ATX inhibitor 2 ccPA has shown promise in treating osteoarthritis,^488^ inflammation, neuropathic pain,^489^ lung fibrosis^490^ and SS.^491^

The class III inhibitor UDCA has been explored as a therapeutic agent against primary biliary cholangitis,^492^ chronic itch,^493^ arthritis,^494^ hepatic and pulmonary fibrosis,^495^ and neurodegenerative diseases such as Parkinson’s disease.^496^ IOA-289 has been investigated for its efficacy in inhibiting cancers with increased fibrosis, such as pancreatic and gastrointestinal cancers^,^^497,498^ as well as fibrotic lung diseases.^499^

GLPG1690 and GLPG ATX inhibitors have been extensively studied for their antifibrotic effects, specifically against IPF and cystic fibrosis, and are in clinical trials.^36^ GLPG1690 has also been reported to have anticancer effects on neuroendocrine, breast, and colorectal cancers.^37,500,501^ BBT-877, another promising ATX inhibitor that is currently in clinical trials for IPF,^43^ has also demonstrated efficacy against DN.^502^ It has demonstrated therapeutic efficacy against DN and OC, where it reduces spheroid formation, metastasis, and therapy resistance.^503^

### LPAR antagonists

In parallel with ATX inhibition, therapeutic strategies targeting LPARs have gained traction across a broad spectrum of diseases, including cancers and cardiovascular, neuroinflammatory, fibrotic, metabolic, and autoimmune disorders. Both small-molecule antagonist and siRNA-based approaches have shown preclinical and clinical efficacy by modulating receptor-mediated signaling.

Selective LPAR antagonists have demonstrated potent effects against multiple pathological processes: the LPAR1 antagonist RO6842262 has been reported to have therapeutic effects against breast cancer,^504^ OC,^505^ liver failure^506^ and lung fibrosis^507^; AM966 attenuates the progression of neuroinflammation and lung fibrosis^245,358^; AM095 inhibits symptoms of scleroderma and diabetic nephropathy^276,373^; BMS-986020 has shown therapeutic effects against IPF, ESCC and ischemic stroke^39,40,392^; SAR100842 has been shown to ameliorate SS conditions^41^; and PIPE-791 has been shown to inhibit neuroinflammation and treat MS.^306^

Dual LPAR1/3 antagonists also hold significant promise: Ki-16425 ameliorates neuropathic pain, inflammation, cardiovascular defects, and numerous cancers.^33,156,508,509^ VPC12249 reduces bone loss, lung fibrosis, and renal IRI,^108,359,384^ and Ki-16198 has shown therapeutic effects against neuropathic pain and pancreatic cancer.^510,511^

Selective antagonists for other LPARs have similarly demonstrated therapeutic efficacy: H2L5186303, an LPAR2 antagonist, inhibited allergic asthma.^512^ The LPAR3 antagonist 8:0 DGPP enhances corneal barrier function and suppresses atherosclerosis^,^^144,513^ whereas VPC51098 attenuates pulmonary fibrosis.^507^

The LPAR5 antagonist H2L5765834 alleviated cancer-associated neuropathic pain^514^; AS2717638 and AS2548635 exhibited analgesic and anti-neuroinflammatory effects^515,516^; and TCLPA5 showed therapeutic potential against psoriasis^517^ and ischemic stroke.^252,253^ The LPAR6 antagonists C75 and XAA suppressed HCC progression.^518^

Notably, the pan-LPAR inhibitor BrP-LPA has demonstrated broad efficacy in suppressing the progression of breast, colon, and ovarian cancers; gliomas; cardiovascular diseases; arthritis; and diabetic retinopathy.^188,519–523^ Several of these antagonists are currently in clinical trials for the treatment of IPF, MS, and SS.

Notably, the therapeutic targeting of LPARs must be tailored to the specific pathological context. While LPAR1 and LPAR3 antagonists have shown efficacy in reducing invasiveness and fibrosis in several models,^1,524^ LPAR6 activation may exert protective effects in certain neurodegenerative or cancer settings.^30,409^ Therefore, precision therapeutic approaches that align with tissue-specific receptor expression profiles and downstream signaling biases will be essential for effective clinical translation.

### Inhibition of downstream targets and combinatorial therapy

In addition to monotherapies that target ATX or LPARs, combinatorial strategies that disrupt multiple components of the LPA axis have demonstrated superior therapeutic efficacy across diverse pathologies. These approaches simultaneously inhibit LPA production, receptor activation, and/or downstream effectors, leveraging the complexity of LPA-driven disease mechanisms.

In cancer models, combining the LPAR1 antagonist Ki16425 with the ATX inhibitor BMP-22 more effectively suppressed melanoma growth and metastasis than either agent alone.^1^ BrP-LPA, a dual ATX–LPAR inhibitor, reduces breast cancer progression by simultaneously blocking LPA synthesis and signaling.^521^ In HCC, pan-ATX-LPAR1 inhibition significantly reduces both hepatic fibrosis and tumor burden, highlighting its utility in fibrotic tumors.^367^ Combination strategies have also been effective in overcoming therapy resistance. Compared with chemotherapy alone, coadministration of the ATX inhibitor ONO-8430506 with paclitaxel improved therapeutic outcomes in both breast cancer and PDAC patients.^525^ Similarly, dual inhibitors 3b and 3 f, which target ATX and LPAR1, effectively eliminate paclitaxel-resistant breast cancer stem cells and reduce melanoma metastasis.^460^ In fibrotic models, dual inhibition with PF-8380 (ATX) and AM-095 (LPAR1) outperformed monotherapies in suppressing bleomycin-induced lung fibrosis.^348^ BrP-LPA also alleviated collagen-induced arthritis via dual inhibition of LPA production and signaling.^484^

Targeting both the LPA and non-LPA pathways has yielded synergistic effects. In NSCLC complicated by IPF, a tetrahydropyrido[4,3-d]pyrimidine compound that targets both EGFR and ATX outperforms traditional EGFR inhibitors such as gefitinib, suggesting a novel strategy for simultaneously managing oncogenic signaling and fibrosis.^526^ In colon cancer, the EZH2 inhibitor GSK126 combined with PF-8380 produced stronger antitumor effects than either agent alone.^527^ In OC models, the use of BBT-877, an ATX inhibitor, along with paclitaxel overcomes paclitaxel resistance in addition to reducing the population of cancer stem cells and the formation of tumor nodes.^503^

Immune-based combinations also show promise. In KRAS/TP53-mutant NSCLC resistant to PD-1 blockade, cotargeting ATX (PF-8380) and PD-1 restored CD8⁺ T-cell infiltration and reversed immune evasion.^438^ Similarly, dual targeting of ATX (PF-8380) and LPAR1 (AM-095) resulted in greater suppression of bleomycin-induced lung fibrosis than monotherapy.^528^

### Current clinical trials targeting the ATX‒LPA‒LPAR axis

The growing preclinical support for targeting LPA signaling has catalyzed the clinical development of both LPAR antagonists and ATX inhibitors. Among the earliest compounds was BMS-986020, a selective LPAR1 antagonist from Bristol Myers Squibb. While it has shown encouraging antifibrotic effects in early trials by slowing declines in lung function and reducing serum biomarkers of fibrosis,^38,40^ the program was terminated because of adverse events such as cholecystitis and hepatotoxicity, emphasizing the critical need for safety monitoring in this drug class.

Another promising LPAR1 antagonist, BMS-986278 (also known as admilparant; NCT04308681), developed by Bristol Myers Squibb, has completed a phase II randomized trial in IPF patients. It demonstrated a favorable safety profile alongside antifibrotic activity in a phase II trial in IPF^529^ and is currently in a phase III trial (NCT06003426), which remains active and recruiting. Similarly, PIPE-791, developed by Contineum Therapeutics, is undergoing phase I evaluation (NCT06683612) in progressive MS and IPF, with trials focusing on pharmacokinetics, tolerability, and early efficacy.

SAR100842, a negative allosteric modulator of LPAR1 that was originally developed by Sanofi for systemic sclerosis, showed tolerability and trends toward clinical benefit in a phase IIa study.^41^ Currently rebranded as HZN-825 (fipaxalparant) under Amgen’s Horizon Therapeutics, it is in phase IIb development (NCT04781543, NCT05032066) for fibrotic diseases.

ATX inhibitors have similarly progressed into clinical development. GLPG1690, developed by Galapagos, initially showed benefits in phase II IPF trials (FLRAs) but was later discontinued in phase III trials (ISABELA 1 and 2) because of safety concerns.^36^ Nevertheless, related compounds in the GLPG series (e.g., GLPG2737, GLPG2222, GLPG2451) have completed early-phase clinical trials with promising results, demonstrating proof-of-concept for modulating LPA signaling in fibrotic lung diseases.^42,530^

BBT-877, developed by Bridge Biotherapeutics, has completed phase I trials assessing safety and tolerability in healthy volunteers and has advanced into phase II studies for IPF.^43,531^ Another notable inhibitor, IOA-289 (NCT05586516), from iOnctura is being evaluated in a phase I/II clinical trial for metastatic PDAC, either alone or in combination with standard chemotherapeutic agents.^497^ Additional ATX-targeting agents include 2 ccPA, which is currently under investigation in phase I/II clinical trials for osteoarthritis sponsored by Orient Europharma. Similarly, BLD-0409 (cudetaxestat) has completed phase I studies for chronic liver diseases and is now in phase II development for IPF, sponsored by Blade Therapeutics.^532^

While challenges in optimizing efficacy and minimizing off-target effects persist, the evolving landscape of ATX–LPA–LPAR inhibitors represents a considerable advance toward precision therapeutics in cancer, fibrosis, autoimmune and inflammatory diseases.

## LPA signaling network: a paradigm for understanding disease complexity

The LPA signaling network exemplifies the intricate complexity of biological systems implicated in a broad spectrum of diseases, including cancer, fibrosis, cardiovascular dysfunction, neurodegenerative disorders, and autoimmune conditions.^1–3,484^ Characterized by receptor redundancy, pathway crosstalk, feedback regulation, and context-specific adaptability, the LPA axis governs essential cellular processes such as proliferation, differentiation, migration, angiogenesis, immune modulation, and metabolic reprogramming.^1,76^ While these features underscore its central role in disease pathogenesis, they also confer adaptive resistance to monotherapies, highlighting the need for systems-level approaches to decode and therapeutically target its complexity.

**Fig. 7:**
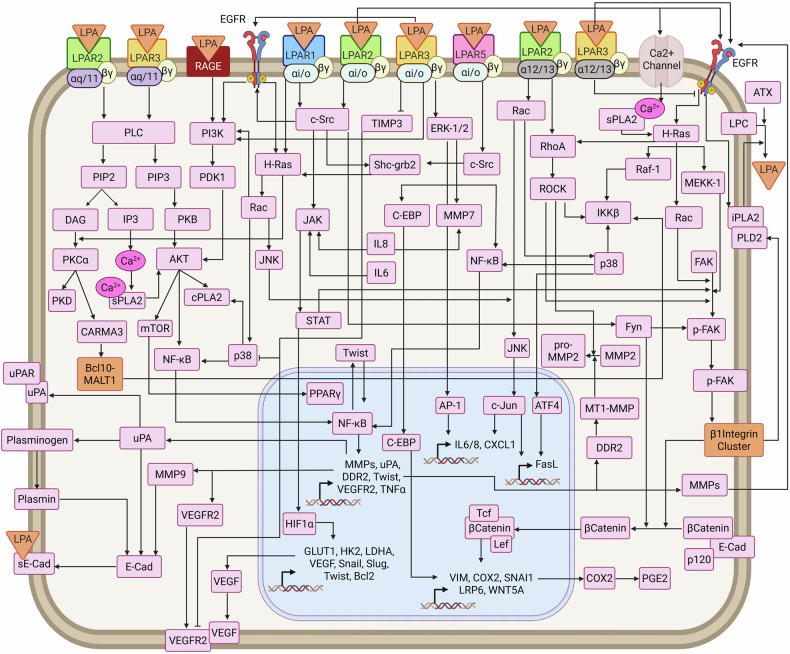
Molecular interaction map of the LPA signaling network in ovarian cancer. This figure illustrates a comprehensive, literature-curated map of the LPA signaling network in ovarian cancer. The signaling molecules are displayed in pink boxes and multi-factor complexes are presented in orange boxes. Nuclear effectors and transcriptional outputs are contextualized to indicate the functional endpoints. The complexity and interconnectivity underscore the need for AI/ML-based modeling approaches to identify actionable signaling hubs and context-specific therapeutic vulnerabilities in LPA-LPAR signaling. Abbreviations for all molecular mediators are provided in the main text. Adapted from the signaling circuit generated using CellDesigner version 4.4.2 (https://www.celldesigner.org/), the figure was created using BioRender.com under an academic license

Among LPA-driven pathologies, OC provides a prototypic system for modeling the network’s signaling intricacies. OC is uniquely suited for this purpose because of the elevated levels of LPA in ascitic fluid, the involvement of multiple LPARs, and compelling evidence linking LPA signaling to hallmark cancer traits, including EMT, angiogenesis, cancer stemness, and chemoresistance. On the basis of this rationale, we constructed a comprehensive molecular interaction map of the LPA signaling circuit in OC (Fig. 7). This network captures key features, redundancy, feedback loops, crosstalk, and signaling heterogeneity, which define the dynamic behavior of LPA signaling in cancer. However, while this static map offers a robust starting framework, it falls short of capturing the temporal dynamics, adaptive rewiring, and emergent behaviors characteristic of LPA-driven disease states.

To overcome these limitations and simulate the real-time complexity of the network, dynamic modeling approaches using artificial intelligence (AI) and machine learning (ML) are essential. Two complementary strategies are envisioned: (1) clinical decision support systems that harness ML to guide personalized treatment decisions on the basis of LPA signaling profiles and (2) mechanistic disease modeling using neural network architectures to predict disease progression, therapeutic response, and resistance mechanisms. Together, these AI-enabled approaches can transform static maps into dynamic, patient-specific predictive engines—paving the way for precision medicine in LPA-associated diseases.

### LPA signaling and neural networks

To justify the use of neural networks in modeling the LPA circuit, it is critical to first recognize the structural and functional similarities between biological signaling systems and AI-based systems. LPA signaling shares several key characteristics with neural networks, making it an ideal candidate for AI-based neural network modeling. Both systems are inherently multimodal, integrating a variety of inputs, such as ligands, cytokines, and environmental cues, through multiple receptors (e.g., LPAR1–6) to generate diverse cellular outcomes.^5,533,534^

They are also multinodal and involve densely interconnected pathways. In the case of LPA signaling, downstream effectors such as PI3K, MAPK, and Rho GTPase orchestrate critical cellular functions, including proliferation, migration, and survival, mirroring the layered architecture of neural networks that process inputs across multiple nodes to derive complex outputs.

Additionally, both systems demonstrate adaptive plasticity, recalibrating in response to internal feedback and external perturbations. Feedback loops and pathway crosstalk enable LPA signaling to dynamically adjust under therapeutic pressure or immune surveillance, paralleling the learning behavior of neural networks.^535–537^

A further point of convergence is context-dependent signal amplification. In cancer cells, LPA signaling can amplify prosurvival and proliferative cues while suppressing apoptotic pathways, analogous to neural networks weighting inputs to optimize outputs on the basis of the training context.^534,537,538^

AI-based models have already proven successful in decoding complex signaling networks such as EGFR, AKT, and ERK, revealing mechanisms of drug resistance and identifying actionable targets.^539–544^ Applying similar frameworks to LPA signaling holds promise for uncovering novel therapeutic vulnerabilities, specifically in ovarian cancer and other LPA-driven diseases.

### Converting the LPA circuit into an interactive model

Transitioning from a static to an interactive model of the LPA signaling network is essential for decoding its full therapeutic potential. Static molecular interaction maps, while foundational, are limited in capturing temporal dynamics, adaptive rewiring, and feedback-based resilience inherent to LPA-regulated systems. In contrast, interactive neural network-based models can simulate how LPA signals are processed, amplified, and modulated under dynamic conditions such as drug treatment, hypoxia, or immune evasion—mimicking cellular adaptation and revealing context-specific therapeutic vulnerabilities.

To construct such a model, critical inputs (e.g., LPA concentrations and receptor expression levels) and outputs (e.g., proliferation, apoptosis, and survival) must be defined. Environmental variables, including the TME, drug exposure, and hypoxia, must be incorporated to enable real-time contextual adaptation, akin to how artificial neural networks prioritize outputs on the basis of evolving stimuli.^541^ Adaptive nodes are essential for replicating biological signal integration; for example, PI3K/AKT signaling may differentially modulate downstream targets such as FOXO1, mTOR, GSK3, TSC2, or MDM2, depending on surrounding signals.^541^ Similarly, feedback loops must be embedded to simulate self-regulating circuit dynamics.

**Fig. 8:**
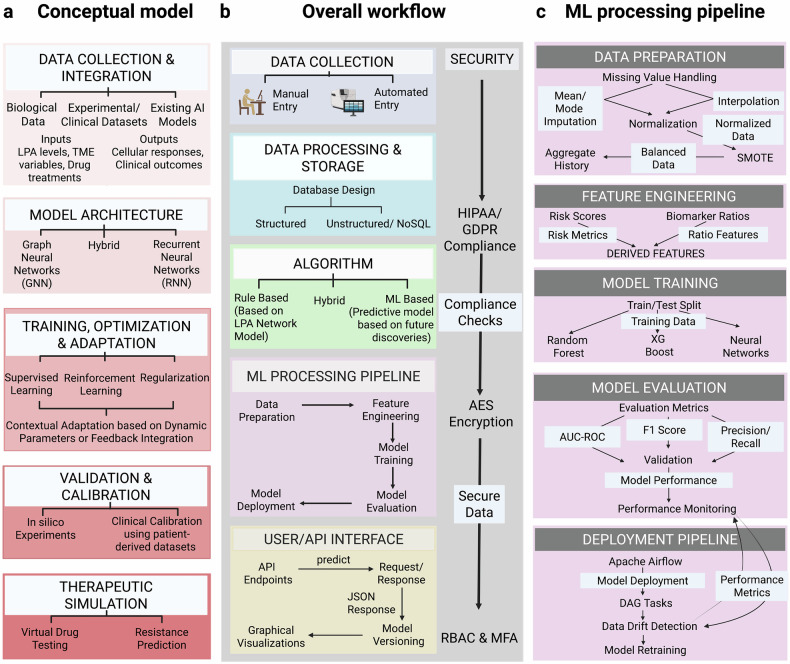
LPA signaling and neural network. This figure outlines the design and implementation strategy for converting the LPA signaling circuitry into an interactive, adaptive AI/ML model for clinical translation. **a** The conceptual model presents the sequential stages involved in building a predictive framework—starting from integration of biological, experimental, and clinical datasets, followed by architecture design (GNN, RNN, or hybrid), contextual learning, calibration using patient data, and culminating in virtual drug testing and resistance prediction. **b** The overall workflow shows the secure pipeline from manual or automated data collection through processing, algorithmic modeling, compliance validation, and deployment, incorporating user interfaces and encryption protocols for clinical scalability. **c** The machine learning (ML) processing pipeline details each step of model development—from data preparation and feature engineering to model training, evaluation, and deployment, including monitoring mechanisms such as drift detection and retraining loops. [AES Advanced Encryption Standard, AI artificial intelligence, API application programming interface, AUC-ROC area under curve – Receiver Operating Characteristic Curve, DAG Directed Acyclic Graph, GDPR General Data Protection Regulation, GNN Graph Neural Network, HIPAA Health Insurance Portability and Accountability Act, JSON JavaScript Object Notation, LPA lysophosphatidic acid, ML machine kearning, MFA multi-factor authentication, NoSQL not only SQL, RBAC role-based access control, RNN recurrent neural network, SMOTE synthetic minority over-sampling technique, TME tumor microenvironment]. The figure was created via BioRender.com under an academic license

This AI-based transformation involves sequential steps: data integration, model architecture design, input–output stratification, training and optimization, contextual calibration, and iterative refinement. Graph neural networks (GNNs), recurrent neural networks (RNNs), or hybrid architectures are particularly suited to model the LPA network, which exhibits layered organization, signaling redundancy, and feedback-driven behaviors (Fig. 8a).

To quantitatively assess the relative contribution of each signaling axis within the LPA network (e.g., MAPK, RhoA-YAP, and PI3K-AKT), our AI modeling approach incorporates several strategies grounded in both deep learning and classical machine learning. Attention-based GNNs, such as graph attention networks (GATs), learn edge weights during training, effectively prioritizing interactions on the basis of their phenotypic impact. These weights effectively represent the model’s estimate of how influential each pathway interaction is in shaping a specific phenotype, such as cell survival, invasion, or chemoresistance. For example, consistently elevated attention scores along the MAPK cascade suggest that this signaling route is particularly determinant in that cellular context. In parallel, classical ensemble models such as random forests or XGBoost decompose predictions into multiple feature-based decision splits. The frequency and depth at which features such as RhoA activity or LPAR6 expression are used across decision trees yield robust feature importance scores, reflecting their predictive power and ranking their biological relevance within the network.

Model interpretability will be further enhanced through the application of SHapley additive exPlanations (SHAPs), which assign each input variable a marginal contribution score on the basis of cooperative game theory. These SHAP values are especially useful in biological systems, where multiple nodes may show correlated activity. For example, if RhoA activity demonstrates high SHAP values across a cohort of samples associated with metastasis, it may play a dominant role in determining disease aggressiveness relative to other nodes, such as PI3K or YAP. To validate model predictions and assess robustness, perturbation-based sensitivity analyses will be incorporated, using either in silico node dropout simulations or experimental data derived from CRISPR/Cas9 and RNAi-mediated knockdowns. These datasets will allow us to examine how removal or suppression of specific components alters predictive outcomes, offering a functional layer of validation. Thus, supervised and reinforcement learning algorithms can be used to train the network, while model adaptability can be improved via iterative learning from experimental feedback and external perturbations. Finally, to explore emergent signaling molecules in the LPA signaling architecture, unsupervised learning methods such as spectral clustering, modularity-based community detection, or node2vec embeddings (unsupervised algorithms for learning network structures) are used. These approaches are anticipated to reveal latent functional modules, collections of coregulated or cooperatively acting nodes, that may represent druggable subnetworks or biomarkers of cellular state transitions.

### Frameworks and validation strategies

To faithfully model the complexity of LPA signaling across diseases, AI frameworks must incorporate nonlinear dynamics, temporal regulation, and embedded feedback mechanisms. RNNs and long short-term memory (LSTM) architectures are particularly adept at simulating time-dependent events such as receptor recycling, transient activation, and oscillatory behavior within signaling networks.

Biological and technical variability across disease models and data sources can be addressed through normalization procedures, domain adaptation, and cross-platform feature harmonization. Additionally, signaling hubs such as PI3K, MAPK, mTOR, and Rho/ROCK can be weighted on the basis of disease-specific relevance to construct predictive models tailored to contexts such as cancer, fibrogenesis, or neuroinflammation. Validation of these AI-modeled circuits must involve robust strategies, including cross-validation with independent datasets, bootstrapping techniques, and perturbation-based assays such as pathway inhibition or gene silencing. These approaches help confirm model fidelity and predictive robustness across diverse biological systems, including cancer and cardiovascular, fibrotic, and neurodegenerative conditions.

Platforms such as TensorFlow and PyTorch, in conjunction with scalable cloud infrastructure (e.g., AWS or GCP), support model development, training, and real-time deployment. Despite challenges related to data heterogeneity, overlapping signaling nodes, and regulatory compliance, this approach enables unsupervised learning, continual model adaptation, and virtual therapeutic testing. Ultimately, the interactive LPA signaling model can serve as a predictive virtual laboratory for exploring disease mechanisms and informing therapy across a wide array of LPA-driven conditions.

### Therapeutic implications of the interactive neural network model

The clinical translation of an AI-powered LPA signaling model hinges on its capacity to identify patient-specific therapeutic targets and optimize precision treatment. Realizing this potential requires a secure, scalable software platform that integrates the LPA model with electronic health records (EHRs) to support data-driven clinical decision-making.

A streamlined implementation architecture includes components for data acquisition, data processing and storage; specific algorithms that can be either rule-based, ML-based or hybrid; an algorithm processing pipeline involving model training and evaluation; model deployment; and an API layer for model versioning and interface development, all of which are performed following tightly regulated security compliance (Fig. 8b). A detailed workflow involving the ML-based processing pipeline is described in Fig. 8c. Languages such as Python (for AI/ML), JavaScript (for front-end interfaces), and SQL/NoSQL (for databases) underpin the system. Frameworks such as TensorFlow or PyTorch enable model development, whereas platforms such as Django or Flask manage back-end processing. Model deployment can be handled through orchestration tools (e.g., Apache Airflow), and cloud environments (e.g., AWS, Azure) ensure scalability and rapid computation. Patient-specific biomarker profiles input via EHR integration or manual entry are normalized and stratified to align with treatment response signatures. Prediction engines may be rule-based or trained on multisource datasets, using statistical metrics such as F1 scores and AUC-ROC to ensure high classification accuracy and clinical relevance. Security and regulatory compliance, including the HIPAA, GDPR, and institutional standards, are essential. Systems must support AES encryption, role-based access control, multifactor authentication, and full audit trials, with flexible deployment on the cloud or onsite infrastructure. An intuitive user interface should visualize biomarkers, treatment predictions, and projected disease trajectories. A human-in-the-loop mechanism allows clinicians to refine AI recommendations, foster adaptive learning and improve decision fidelity with each iteration.

The neural frameworks, GNNs, RNNs, and reinforcement learning enable the simulation of complex biological interactions and adaptive resistance. The integration of multiomics data allows the mapping of network bottlenecks and emergent vulnerabilities. In OC, this model helps differentiate sensitive from resistant phenotypes and informs combination therapies to bypass LPA-driven chemoresistance. As a next-generation enhancement, the interactive LPA model could evolve into a digital twin, a dynamic, patient-specific simulation of LPA network behavior, continuously updated with molecular, clinical, and therapeutic data. This twin could predict disease evolution, test virtual drug regimens, and optimize intervention timing in real time. Although OC has been demonstrated, this architecture is broadly applicable to LPA-driven pathologies, including fibrosis, cardiovascular disease, metabolic syndromes, autoimmune conditions, and neurodegeneration. As such, AI-based LPA modeling represents a transformative advance in applying systems biology to precision medicine.

## Perspectives and future directions

The lysophosphatidic acid (LPA) signaling axis, which includes autotaxin (ATX), LPA, its receptors (LPARs), and downstream effectors, has emerged as a critical regulatory network across diverse diseases, from cancer and fibrosis to cardiovascular, neurological, and autoimmune disorders. This axis orchestrates key pathological processes such as cell proliferation, invasion, angiogenesis, immune evasion, and therapy resistance, making it a compelling target for precision therapeutic strategies.

Pharmacological targeting of the LPA pathway continues to advance, as LPAR1 antagonists such as BMS-986020 (NCT01766817) and BMS-986278 (NCT04308681) have shown antifibrotic efficacy in pulmonary disease. Similarly, the ATX inhibitor GLPG1690, initially developed for idiopathic pulmonary fibrosis (IPF), reinforces the feasibility of modulating this signaling cascade. Combination strategies that target both LPA synthesis and receptor-mediated signaling may provide superior efficacy by disrupting compensatory feedback mechanisms and network redundancy. In addition to drug development, computational and AI-driven approaches present new opportunities to understand and manipulate the LPA network at the systems scale. These models move beyond static pathway representations, offering dynamic simulations that adapt to real-time inputs and patient-specific variables. By incorporating architectures such as graph neural networks and reinforcement learning, these systems can refine therapeutic strategies on the basis of evolving disease signatures and feedback loops.

Given its diverse receptor expression and pleiotropic signaling roles, LPA sits at the intersection of nervous system regulation, immune activation, and metabolic control, underscoring its role as a systems-level hub and a promising target for systems biology-guided targeted therapy in complex diseases such as neurodegeneration, autoimmune disorders, cardiovascular disease, and cancer. Future implementations should integrate these AI frameworks with clinical infrastructures, enabling real-world application through electronic health records and decision support platforms. One such frontier is the concept of digital twins, previously discussed, wherein patient-specific virtual models evolve with integrated multiomics and clinical data. These tools have the potential to predict therapeutic responses, identify resistance mechanisms, and personalize interventions in real time. To scale the impact of LPA network modeling, future directions may include leveraging real-world evidence (RWE) and population-level datasets for risk stratification and treatment optimization. Furthermore, embedding AI models into clinician-facing systems with interpretable outputs and colearning capabilities may accelerate adoption and trust in AI-mediated precision medicine. In conclusion, the LPA signaling network represents both a complex biological enigma and a transformative therapeutic opportunity. As we move toward AI-enabled precision medicine, decoding the LPA network’s dynamic behavior across diseases may unlock novel strategies to improve outcomes in an array of pathologies driven by its dysregulation.

## Data Availability

This manuscript does not report data generation or analysis.
